# Functional mapping of androgen receptor enhancer activity

**DOI:** 10.1186/s13059-021-02339-6

**Published:** 2021-05-11

**Authors:** Chia-Chi Flora Huang, Shreyas Lingadahalli, Tunc Morova, Dogancan Ozturan, Eugene Hu, Ivan Pak Lok Yu, Simon Linder, Marlous Hoogstraat, Suzan Stelloo, Funda Sar, Henk van der Poel, Umut Berkay Altintas, Mohammadali Saffarzadeh, Stephane Le Bihan, Brian McConeghy, Bengul Gokbayrak, Felix Y. Feng, Martin E. Gleave, Andries M. Bergman, Colin Collins, Faraz Hach, Wilbert Zwart, Eldon Emberly, Nathan A. Lack

**Affiliations:** 1 Vancouver Prostate Centre, Department of Urologic Science, University of British Columbia, Vancouver, Canada; 2 School of Medicine, Koç University, Istanbul, Turkey; 3 Koç University Research Centre for Translational Medicine (KUTTAM), Koç University, Istanbul, Turkey; 4 Department of Physics, Simon Fraser University, Burnaby, Canada; 5 Division of Oncogenomics, Oncode Institute, The Netherlands Cancer Institute, Amsterdam, The Netherlands; 6 Division of Molecular Carcinogenesis, Oncode Institute, The Netherlands Cancer Institute, Amsterdam, The Netherlands; 7 Division of Urology, The Netherlands Cancer Institute, Amsterdam, The Netherlands; 8 Helen Diller Family Comprehensive Cancer Center, University of California, San Francisco, USA; 9 Division of Medical Oncology, The Netherlands Cancer Institute, Amsterdam, The Netherlands; 10 Department of Biomedical Engineering, Eindhoven University of Technology, Laboratory of Chemical Biology and Institute for Complex Molecular Systems, Eindhoven, The Netherlands

**Keywords:** Prostate cancer, Androgen receptor, Enhancers, STARRseq, Non-coding mutations

## Abstract

**Background:**

Androgen receptor (AR) is critical to the initiation, growth, and progression of prostate cancer. Once activated, the AR binds to cis-regulatory enhancer elements on DNA that drive gene expression. Yet, there are 10–100× more binding sites than differentially expressed genes. It is unclear how or if these excess binding sites impact gene transcription.

**Results:**

To characterize the regulatory logic of AR-mediated transcription, we generated a locus-specific map of enhancer activity by functionally testing all common clinical AR binding sites with Self-Transcribing Active Regulatory Regions sequencing (STARRseq). Only 7% of AR binding sites displayed androgen-dependent enhancer activity. Instead, the vast majority of AR binding sites were either inactive or constitutively active enhancers. These annotations strongly correlated with enhancer-associated features of both in vitro cell lines and clinical prostate cancer samples. Evaluating the effect of each enhancer class on transcription, we found that AR-regulated enhancers frequently interact with promoters and form central chromosomal loops that are required for transcription. Somatic mutations of these critical AR-regulated enhancers often impact enhancer activity.

**Conclusions:**

Using a functional map of AR enhancer activity, we demonstrated that AR-regulated enhancers act as a regulatory hub that increases interactions with other AR binding sites and gene promoters.

## Background

Androgen receptor (AR)-mediated transcription is the primary driver of prostate cancer (PCa) growth and proliferation [1]. Activation of this critical signaling pathway occurs when AR binds to androgens such as testosterone or dihydrotestosterone (DHT). This induces the translocation of the AR into the nucleus, where it interacts with DNA at AR binding sites (ARBS). Almost all of these *cis*-regulatory elements (CREs) are located at distal intergenic or intronic regions [2, 3]. The AR cistrome is influenced by various transcription factors and pioneer factors, including FOXA1, HOXB13, and GATA2 [2, 4, 5]. Once bound to DNA, the AR recruit numerous co-activators (CBP/p300, SRC/p160), chromatin modifiers (SWI/SNF-BRG1), and co-repressors (HDAC, NCoR) in a highly coordinated manner [6]. This protein complex physically interacts with gene promoters via chromosomal loops, activating basal transcriptional machinery to drive transcription. Yet similar to other nuclear receptors, most AR-regulated genes interact with multiple ARBS [7]. There are vastly more ARBS (tens of thousands) than AR-regulated genes (hundreds) [8, 9]. We do not know if these ARBS enhancers interact in an additive, synergistic, or dominant mechanism to induce gene transcription. Characterization of ARBS enhancer activity is critical to interpret the underlying regulatory logic of this transcription factor.

Enhancers have traditionally been identified by correlating transcription factor binding sites with chromatin accessibility, RNA polymerase II, GROseq, or enhancer-associated histone modifications such as H3K27ac [10–13]. These features all broadly correlate with active enhancers, but they are not causative and therefore are extremely prone to false positives [14]. For example, global loss of the enhancer mark H3K27ac has no functional impact on gene transcription, chromatin accessibility, or histone modifications [15]. Therefore, ectopic reporter assays, which quantify the enhancer-induced transcription of a gene, still remain the cornerstone of enhancer validation [16]. These assays are not influenced by endogenous chromatin compaction or epigenetic modifications and can test the potential enhancer capability of each specific CRE [17]. While robust, conventional approaches are very low-throughput. To overcome these limitations, several massively parallel reporter assays (MPRA) have been developed including Self-Transcribing Active Regulatory Regions sequencing (STARRseq) [18]. In this method, enhancer activity is quantified by measuring the rate of self-transcription of the genomic region cloned downstream of a minimal promoter. By quantifying self-transcribed mRNA, the enhancer activity of many thousands of potential regulatory sites can be measured simultaneously and provide locus-specific resolution.

There is increasing clinical evidence that non-coding mutations can act as oncogenic drivers in PCa [19–21]. Recent studies by our lab and others have shown that ARBS are highly mutated in a tissue-specific manner [22, 23]. Given the critical role of AR in PCa progression and treatment resistance, any changes to the transcriptional landscape could alter tumor cell proliferation and sensitivity to AR pathway inhibitors. However, establishing a causal link between non-coding mutations and PCa growth is extremely challenging due to the lack of functional CRE annotation. Therefore, the vast majority of these non-coding mutations remain unexplored in PCa. Better characterization of these CRE in PCa is essential to stratify potential driver mutations.

To provide the first locus-specific AR regulatory map, we functionally quantified the enhancer activity of all commonly observed clinical ARBS with STARRseq. We demonstrated that only 7% of ARBS have androgen-dependent enhancer activation, while 11% had enhancer activity that was independent of AR binding. Surprisingly, the vast majority of ARBS (81%) did not have significant androgen-dependent or constitutively active enhancer activity. These in vitro annotations strongly correlated with enhancer associated histone modifications in clinical PCa samples. To characterize the mechanism of AR enhancers, we then trained a machine learning classifier that successfully predicted active enhancers and identified key features of active enhancers. Integrating both the long-range chromatin interactome and transcriptomic data, we found that androgen inducible enhancers were significantly more enriched as anchors for gene looping and acted as “hubs” to activate AR-regulated genes. Finally, combining these results with whole genome sequencing of primary and metastatic PCa, we identified and characterized a non-coding somatic mutation that significantly impacted AR enhancer activity of a critical tumor suppressor.

## Results

### Functional quantification of AR enhancer activity

**Fig. 1:**
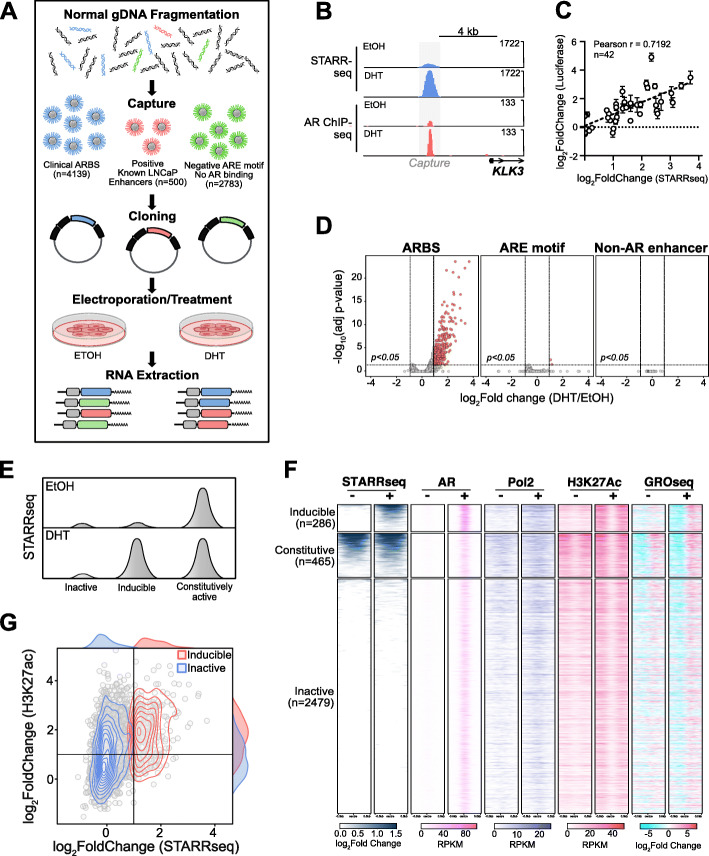
STARRseq identifies AR-dependent enhancers. **a** Schematic representation of AR STARRseq. In this, high confidence ARBS (*n* = 4139), non-AR enhancers (positive control; *n* = 500), and regions with ARE motifs but no AR binding (*n* = 2783) were captured from normal human DNA and cloned into hSTARR-ORI plasmid. The resulting plasmid library was transfected into LNCaP cells by electroporation. Following DHT/EtOH treatment, STARR mRNA was extracted and sequenced to quantify the enhancer-mediated rate of self-transcription at each region. **b** Strong androgen-dependent enhancer activity (blue) was observed at known AR binding sites (red; GSE83860) proximal to *KLK3*. **c** Enhancer activity of AR CREs with varying levels of STARRseq signal (*n* = 42) was validated with a luciferase assay (4 biological replicates ± SEM). A strong correlation is observed between luciferase and STARRseq signals. **d** Volcano plot of androgen-dependent changes in STARRseq enhancer activity for clinical ARBS, ARE motif alone, and non-AR enhancers. Significantly induced enhancers (LFC > 1, *p-*adj < 0.05) are highlighted in red. **e** Schematic representation of the different classes of AR enhancers. **f** Heatmap of STARRseq (blue) represented as LFC over input plasmid library. Publicly available ChIPseq of AR (GSE83860, pink), Pol2 (GSE28126, purple), and H3K27ac (GSE51621, pink) in EtOH or DHT-treated LNCaP cells is shown as reads per kilobase of transcript, per million mapped reads (RPKM). GROseq (GSE83860) shows the normalized LFC of either the positive (pink) or the negative (cyan) RNA strands. The heatmap is divided based on the functional classes of each enhancer class identified by STARRseq. **g** Density map of androgen-induced changes to H3K27ac ChIPseq and STARRseq at inactive and inducible AR enhancers

To characterize AR CREs, we experimentally tested the enhancer activity of all commonly occurring clinical ARBS with STARRseq, a massive parallel enhancer assay (Fig. 1a). In this approach, genomic DNA is cloned downstream of a minimal promoter. Those sites with high enhancer activity will cause high self-transcription that is expressed as mRNA. By quantifying the relative rate of self-transcribed RNA with next-generation sequencing, the enhancer activity can be directly measured. During optimization of this method, we found that similar to published work [24], smaller ARBS inserts (< 250 bp) had lower STARRseq activity, suggesting that the flanking sequences contribute to AR enhancer activity (Additional file 1: Fig. S1). As the current synthesis limit of pooled oligos is ~ 200 bp, we used a capture-based approach to maintain a large insert size. To avoid testing rare or poor-quality ARBS, we targeted those clinical AR peaks identified from a large ChIPseq study [2] that were found in either all normal prostate tissue (*n* = 7), primary PCa tumors (*n* = 13), or both. With this conservative selection criterion, we identified 262 ARBS present in only normal prostate tissue, 3225 ARBS present only in PCa tumors, and 652 ARBS in both normal and PCa tumors. Having selected these regions, we designed a custom DNA capture assay to enrich three different groups: common clinical ARBS (clinical ARBS; *n* = 4139), a positive control of previously identified strong enhancers that are not associated with AR [25] (*n* = 500), and regions that contain an androgen response element (ARE) motif but there is no AR binding in either clinical samples or cell lines (*n* = 2783). With this, we then captured fragmented normal genomic DNA and cloned it into a second-generation STARRseq plasmid [26]. A total of 365,265 unique on-target inserts (median 50 inserts/region) were cloned, with a normal distribution of inserts across our capture regions and a median insert size > 500 bp (Additional file 1: Fig. S2A). Using this targeted library, we tested for AR enhancer activity in an androgen-dependent PCa cell line (LNCaP). The resulting data demonstrated good reproducibility across biological replicas (*Pearson correlation 0.84–0.99*; Additional file 1: Fig. S2B) and a strong STARRseq signal at known AR enhancers including the AREIII that regulates *KLK3* (Fig. 1b). To validate our STARRseq results, we randomly selected ARBS consisting of high, intermediate, and low enhancer activity (*n* = 42) and tested with a luciferase reporter assay. Similar to previously published work, our results for the STARRseq enhancer activity correlated with a conventional luciferase reporter assay [18, 26] (Fig. 1c). Importantly, as STARRseq is a plasmid-based approach, the enhancer activity is independent of endogenous chromatin compaction and therefore quantifies the potential activity at each genomic region. This is clearly demonstrated with the non-AR positive controls which had strong enhancer activity regardless of being found in either heterochromatin or euchromatin [25] (Additional file 1: Fig. S3). When comparing all genomic regions tested, AR-driven enhancer activity was almost exclusively limited to clinical ARBS with only 2/2783 ARE motif containing regions showing a significant increase in signal following androgen treatment (Fig. 1d). Interestingly, within the clinical ARBS regions, we observed three distinct classes of enhancer CRE: a “classical” AR enhancer that increases activity when treated with androgen (inducible), enhancers that were active regardless of androgen treatment (constitutive), and those ARBS that had minimal enhancer activity (inactive) (Fig. 1e). Of these, inactive ARBS were by far the most common (81.9%; 3388/4139) with no significant enhancer activity either before or after ARactivation. A total of 11.2% (465/4139) and 6.9% (286/4139) were constitutively active or inducible enhancers, respectively. While there is significant overlap in the AR cistrome of LNCaP and the tested clinical binding sites, some ARBS are unique to only clinical PCa and not found in LNCaP cells. Interestingly, none of the clinical specific ARBS were inducible enhancers (*n* = 867; Additional file 1: Fig. S4A + B). This rate of inactivity is significantly less than expected compared to the clinical ARBS that overlap with LNCaP (*p* < 2.1 × 10^−16^). As these regions did not have AR binding in our experimental model, they were separated in subsequent analysis (no AR). To confirm that our AR CRE annotations correlated with enhancer activity in vitro, we compared each group with published enhancer-associated features including H3K27ac, RNA polymerase II (Pol2), and bidirectional eRNA (GROseq) [27–29] (Fig. 1f). We observed a strong correlation between the enhancer groups and these features. Specifically, constitutive AR enhancers demonstrated high levels of H3K27ac, Pol2, and eRNA that were comparable in both androgen-deprived (EtOH) and androgen-containing (DHT) conditions. In contrast, these features increased for inducible enhancers when cells were treated with androgens. For inactive ARBS, enhancer-associated features were broadly reduced compared to active enhancers though there was some variation observed. We also found that there were marked differences in AR-mediated DNase I hypersensitive sites between different ARBS, with inducible enhancers increasing accessibility following either 4 or 12 h of androgen treatment (Additional file 1: Fig. S5A). There was no significant enrichment for any one AR enhancer class at super enhancers as these elements are relatively rare at ARBS (Additional file 1: Fig. S5B). Yet while such descriptive features generally correlate with active AR enhancers, they are extremely prone to false-positives at individual CRE. For example, while inducible AR enhancers generally have higher androgen-induced H3K27ac than inactive ARBS, there is significant overlap between these classifications (Fig. 1g). Given that inactive CREs are far more common than induced enhancers, this dramatically increases the false-positive rate. Specifically, if an active enhancer is called solely on AR and H3K27ac ChIPseq, there is a > 80% false-positive rate. Supporting these results, the enhancer activity of high H3K27ac inactive and inducible CRE was validated with a luciferase reporter assay (Additional file 1: Fig. S6). Overall, these results demonstrate that functional enhancer testing with STARRseq can provide locus-specific resolution that is needed to annotate AR CREs.

### Clinical validation of enhancer annotation

**Fig. 2:**
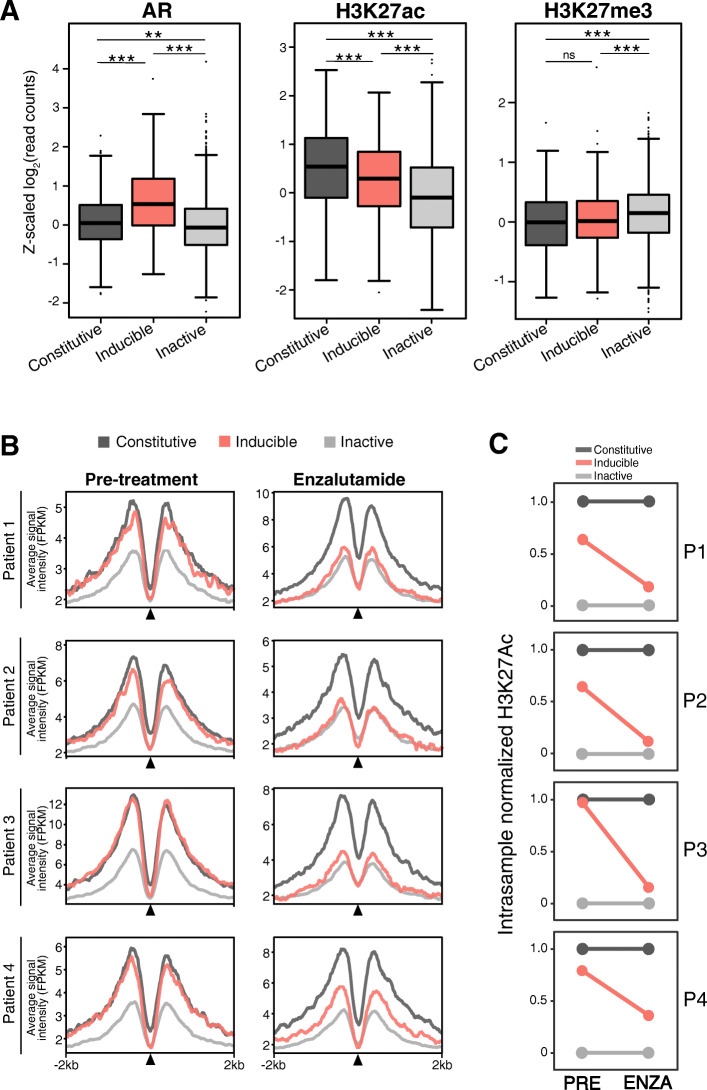
In vitro enhancer classification is preserved in clinical samples. **a** Normalized ChIPseq of AR (*n* = 87), H3K27ac (*n* = 92), and H3K27me3 (*n* = 76) from primary PCa samples. A significant enrichment of AR and H3K27ac is observed at inducible and constitutive ARBS compared to inactive enhancers (*ns* > 0.05, **p < 10^−4^, ***p < 10^−6^). H3K27me3 was also significantly enriched at inactive enhancers compared to inducible or constitutive enhancers. **b** H3K27ac ChIPseq was done in 4 patients with matched PCa tissue pre- and post-enzalutamide treatment. The box plots show the normalized H3K27ac enrichment ±2 kb around induced (red), constitutive (dark gray), and inactive (gray) ARBS enhancers. **c** H3K27ac enrichment in each class of enhancers was normalized within each tumor and compared before and after ENZA treatment. H3K27ac enrichment at induced enhancers was markedly reduced after ENZA treatment

While histone modifications do not accurately identify individual AR enhancer CREs (Fig. 1g), these features, particularly H3K27ac, do broadly correlate with active enhancers (Fig. 1e). Therefore, to determine if our enhancer annotations represent clinical AR activity, we analyzed previously published AR (*n* = 87), H3K27ac (*n* = 92), and H3K27me3 (*n* = 76) ChIPseq from primary PCa tissue [8]. Supporting our in vitro classifications, we observed significant enrichment of both AR and H3K27ac at inducible and constitutive enhancers as compared to inactive ARBS (*p-adj* < 0.0001; Fig. 2a). Further, while not as dramatic, we also found a statistically significant enrichment of the repressive H3K27me3 mark at inactive ARBS compared to constitutive and induced enhancers (*p-adj* < 0.05). However, as these primary PCa samples contain physiological levels of androgen, we could not separate induced and constitutive enhancers as the AR would be active in these tumors. Therefore, to further validate our in vitro classifications, we conducted H3K27ac ChIPseq on prostate tumors from patients enrolled in a neoadjuvant antiandrogen enzalutamide (ENZA) clinical trial (NCT03297385). ENZA is a classical antagonist that directly inhibits androgen binding and prevents translocation of the AR into the nucleus [30]. Therefore, by characterizing the H3K27Ac in matched tumor samples collected pre- and post-ENZA, we can interrogate the impact of AR activity on H3K27Ac. As expected, ChIPseq results from pre-ENZA patients were very similar to the primary PCa samples with an enrichment of H3K27ac in constitutive and inducible ARBS as compared to inactive ARBS (Fig. 2b). However, when the AR is inhibited by ENZA treatment, H3K27ac was enriched only at constitutive enhancers while both inducible and inactive CRE had markedly lower histone modifications (Fig. 2b). When normalized to constitutive and inactive CRE, inhibiting the AR in these patients strongly reduced H3K27ac at inducible AR enhancers (Fig. 2c). Overall, these results demonstrate that our in vitro classifications strongly correlate to clinical AR activity and suggest that this plasmid-based enhancer assay represents AR activity in situ*.*

### Genomic features associated with AR enhancers

**Fig. 3:**
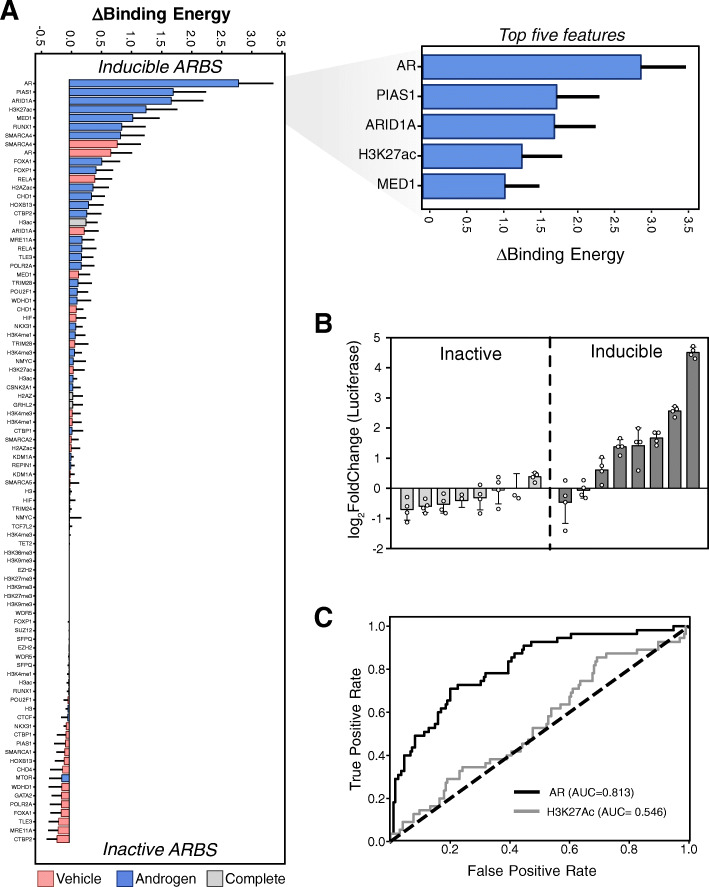
Identification of features associated with AR enhancers. **a** Features from all publicly available transcription factor and histone mark ChIPseq datasets in LNCaP cells (GEO accession and the citations provided in supplementary data) were uniformly processed and binding energy was calculated. The bar graph shows the features sorted by their binding energy at inducible enhancers. The zoomed section (top right) shows the top 5 features that are predictive of inducible enhancers. **b** LNCaP ARBS predicted by the machine learning classifier as either inactive or inducible enhancers were validated by luciferase assay (4 biological replicates±SEM). **c** Receiver operating characteristic curve of AR and H3K27ac ChIPseq to accurately identify inducible enhancers

Having mapped the AR CRE enhancer activity, we next analyzed the DNA motifs at each ARBS to determine what feature correlated with active enhancers. Unfortunately, this gave very poor results with almost no difference in DNA motifs including AREs at inactive, inducible, and constitutively active ARBS (Additional file 1: Fig. S7A). This matches with our experimental findings, where almost no genomic regions that only contain an ARE motif alone and no AR binding had inducible enhancer activity (Fig. 1d). To better characterize these CREs, we then incorporated all publicly available ChIPseq data from LNCaP (*n* = 90; Additional file 2) and trained a machine learning classifier to predict enhancer activity at each ARBS (Fig. 3a). All transcription factor and histone ChIPseq were processed and normalized with a standardized bioinformatic pipeline to reduce technical variation. With this extremely large experimental dataset, we utilized a bootstrapped multinomial logistic regression model with a sparsity LASSO regularizer to identify inducible and inactive ARBS. On test data, our model managed 65% precision for the inducible group and a 62% precision for the inactive group with an overall accuracy of 60%. Given the low frequency of induced enhancers, this is a > 10× enrichment compared to random ARBS. The introduction of additional functional genomic datasets including DHS or ATACseq did not improve the predictive power of this model. To validate this model, we experimentally tested LNCaP-specific ARBS not included in our clinical STARRseq library that were predicted to be inducible (*n* = 8) or inactive (*n* = 8) enhancers (Fig. 3b). Confirming the model, we observed that ~ 60% of the predicted induced enhancers could be accurately identified with our classifier. As this uses a relatively simple multinomial logistic regression model, we can quantify the predictive strength of each DNA-bound factor and identify those features that strongly correlate with inducible AR enhancers. When calculating the differential binding energy for the inducible and inactive groups, we observed that most features associated with inducible enhancers were unsurprisingly found in androgen-treated conditions. Specifically, AR, PIAS1, ARID1A, MED1, and RUNX1 binding in androgen-treated conditions were strong predictors of inducible AR enhancers (Fig. 3a). In contrast, occupancy by CTBP2, WDHD1, and TLE3 at ARBS in EtOH were generally predictive of inactive CRE though these did not have comparable predictive strength to inducible features. To identify which functional genomic features best predicted inducible AR enhancers, we down-sampled our model by reducing the number of features and then re-tested each classifier compared to the general model. With this, we found that of all individual features, AR + DHT peak height had the best power to identify inducible enhancers at ARBS and was significantly better than either H3K27ac or any other transcription factors/histone marks (Fig. 3c; 0.81 vs. 0.55 AUC). If expanded to three features, ChIPseq of AR+/−DHT and PIAS1 + DHT gave the best results with comparable recall to the larger general model (Additional file 1: Fig. S7B; 0.83 vs. 0.86 AUC). Overall, this machine learning classifier provides a powerful tool to identify both those regions that are likely to be inducible enhancers and also the specific features associated with active AR enhancers.

### Role of AR enhancers on gene expression

**Fig. 4:**
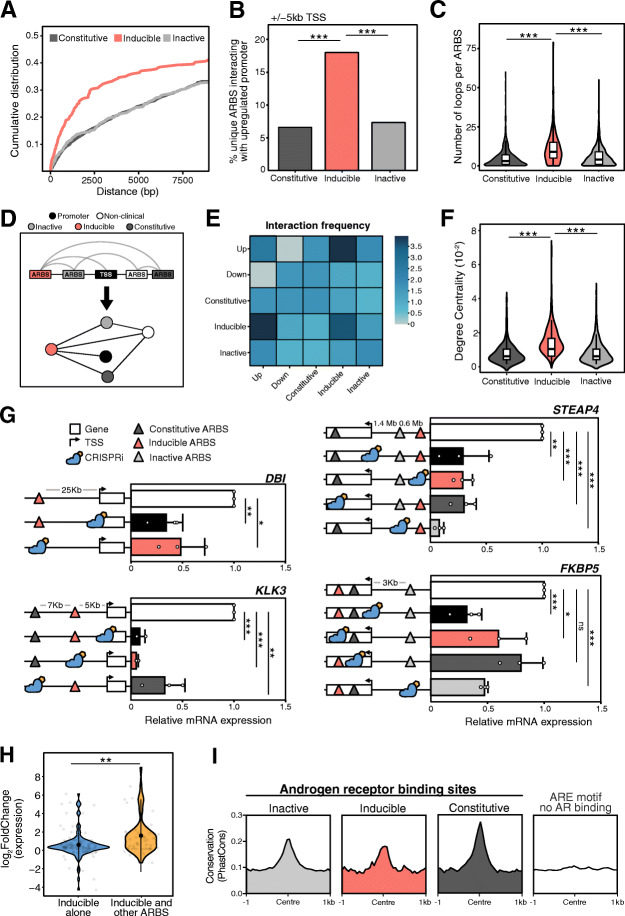
Transcriptional regulation by AR enhancers. **a** Cumulative distribution function correlating the distance (bp) of each enhancer class to the promoters of androgen-upregulated genes. **b** Chromatin loops at ARBS (VCaP ChIA-PET) were overlapped with the enhancer classifications to identify those ARBS that looped to a promoter (± 5 kb from the TSS) of an androgen-upregulated gene. **c** The violin plots shows the number of AR chromatin interactions in each enhancer class. **d** Schematic representation of AR ChIA-PET data transformed into graph network. **e** Calculation of the relative interaction frequency in the graph network between androgen-upregulated gene promoters (Up), androgen-downregulated gene promoters (Down), and each ARBS enhancer class. **f** With the interaction graph network, the betweenness centrality in the largest connected graph was calculated for each enhancer class (*ns p* > 0.05, ***p < 10^−9^). **g** At AR-regulated genes, individual ARBS CRE (induced, inactive, constitutive) were inhibited with CRISPRi (blue) in LNCaP cells to determine their impact on AR transcription. Gene expression was quantified by qPCR and normalized to non-targeting gRNA controls (white bar). The TSS of each gene was also targeted with CRISPRi as a positive control (black bar) (3 biological replicates ± SD;***p < 10^−9^). **h** Androgen-induced expression of genes regulated by only inducible enhancers (*n* = 102) or both inducible enhancers and other ARBS (*n* = 58) (**p < 10^−4^). **i** Evolutionary conservation from 100 vertebrate species of different ARBS enhancer classes compared to genomic regions with ARE motif but no AR binding (*n* = 2783)

We next investigated how each AR enhancer class impacts androgen-mediated transcription. As enhancer-promoter (E-P) interactions frequently occur within neighboring primary sequences [24, 31], we first correlated the distance between promoters of androgen-induced genes to each AR enhancer class. We observed that inducible enhancers were significantly more likely to be near an androgen-upregulated differentially expressed genes (DEG) than a constitutive or inactive enhancer (Fig. 4a). However, E-P interactions frequently occur over considerable distances in the primary sequence due to chromatin looping [32]. Therefore, to quantify the chromatin loops between individual ARBS and AR-regulated genes, we incorporated a published AR ChIA-PET dataset from VCaP cells. While AR expression is higher in VCaP than LNCaP cells, we observed a good congruence in both the AR cistrome and H3K27ac induction at functionally annotated clinical ARBS (Additional file 1: Fig. S8). With this chromatin looping data, we found that inducible ARBS interact more frequently with promoters at androgen upregulated DEG than either constitutive or inactive ARBS (*p* < 0.0001; Fig. 4b). No enrichment was observed between any enhancer class and androgen downregulated DEG suggesting that this occurs through an indirect mechanism. We also observed that inducible AR enhancers form significantly more chromatin loops to either other ARBS or chromosomal sites suggesting that they may act as regulatory “hubs” (*p* < 2 × 10^−16^; Fig. 4c). To better quantify the relationship between AR CREs, we transformed the pairwise interactions into an undirected network with all LNCaP ARBS or DEG TSS being represented as a vertex and chromatin loops as edges (Fig. 4d). All ARBS were included, even those not tested by STARRseq, to provide a comprehensive AR interaction network. Matching our earlier analysis, we found that inducible AR enhancers have a significantly enriched interaction frequency with upregulated DEG (Fig. 4e). When quantifying the relationships between AR CREs in connected or independent networks, we found that inducible enhancers were significantly more likely to be a central node in this regulatory network (*p* < 2 × 10^−9^; Fig. 4f, Additional file 1: Fig. S9A). A similar trend was observed when comparing networks of only tested ARBS regions (Additional file 1: Fig. S9B). These findings suggest that inducible enhancers may play a critical role in AR-mediated transcription as a central node between promoters and AR CREs.

To functionally test these descriptive results, we used CRISPRi to selectively inhibit annotated CREs and quantify the impact on AR-mediated gene expression [33]. Genes were chosen based on their enhancer complexity ranging from a single inducible enhancer that loops to the gene promoter (*DBI*) to a mixture of inactive, constitutive, and inducible AR enhancers (*KLK3*, *STEAP4*, *FKBP5*) (Fig. 4g; Additional file 1: Fig. S10A). Similar to previously published work, when the *KLK3* inducible AR enhancer (AREIII) was inactivated, it also decreased the neighboring gene *KLK2* [34]. Highlighting the specificity of this approach, inactivation of the *KLK3* promoter did not downregulate *KLK2* (Additional file 1: Fig. S10B). In support of our functional assay, the inactivation of inducible AR enhancers significantly reduced androgen-mediated transcription at all genes tested (Fig. 4g). Surprisingly, in addition to the inducible enhancers, many of the constitutive and inactive ARBS also contributed to AR-mediated gene transcription. Inhibition of the *KLK3* constitutive enhancer significantly impaired gene expression suggesting a co-operative action between these two enhancers. In contrast, inhibition of a constitutive enhancer did not significantly alter androgen-mediated transcription of *FKBP5,* while targeting either the AR inducible enhancer or inactive ARBS reduced expression. Finally, AR-mediated transcription of *STEAP4* was significantly reduced by inhibiting inactive, constitutive, or inducible ARBS. This is particularly striking as the *STEAP4* inducible AR enhancer forms a chromosomal loop to this TSS over a > 2 Mb distance (Additional file 1: Fig. S10A). These functional results suggest that while inducible enhancers are critical for gene expression, other AR enhancer classes can also contribute to gene transcription. Supporting this role, we observed that genes regulated by inducible enhancers that interact with inactive or constitutive enhancers had significantly higher levels of androgen-mediated transcription than those inducible enhancers that do not (*p* < 0.0001; Fig. 4h). To confirm this was not an artifact of the experimental system, we next quantified the evolutionary conservation of each ARBS class. This was based on the assumption that only those CREs involved in gene transcription would be conserved via selective pressure. In support of our in vitro data, we observed similar evolutionary conservation of inducible, inactive, and constitutive ARBS that were significantly higher than random ARE motif regions (Fig. 4i). Overall, this data suggests that while inducible ARBS have increased contacts with both promoters and other ARBS, both inactive and constitutive enhancers can play a role in gene transcription.

**Fig. 5:**
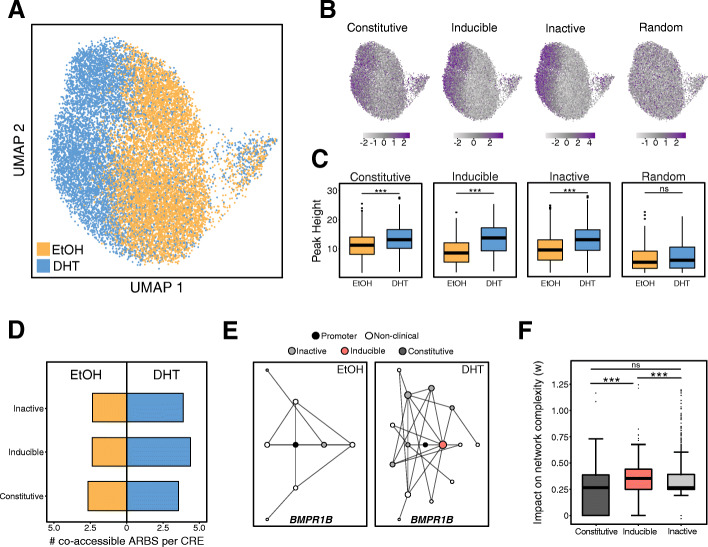
Inducible enhancers are regulatory hubs. **a** Uniform Manifold Approximation and Projection (UMAP) of scATACseq profiles of LNCaP cells treated with either EtOH or DHT. Each dot represents an individual cell (EtOH *n* = 7857; DHT = 6661). **b** chromVAR deviation score enrichment of different AR enhancer classifications was compared to random genomic regions in the UMAP. **c** The chromatin accessibility of pseudo-bulk scATACseq at each AR enhancer class compared to random genomic regions. **d** Change in the median number of co-accessible sites for each AR enhancer class following androgen treatment. **e** A representative network graph of the CREs of *BMPR1B* shows changes in co-accessibility following androgen treatment. The gained inducible AR enhancer led to significant increases in network complexity. **f** The relative impact on network complexity following DHT treatment was calculated for all ARBS in each AR enhancer class. Inducible enhancers had the most significant impact on network complexity leading to higher co-accessibility with other ARBS (*p* < 10^−6^)

Given these results, we proposed that inducible enhancers may act as a regulatory hub between multiple ARBS and gene promoters. To test this, we conducted single cell ATACseq (scATACseq) to identify co-accessible AR CREs and determine the *cis*-regulatory interactions (Additional file 1: Fig. S11). Co-accessible DNA elements strongly correlate with physical proximity [35] and can characterize how AR binding changes CRE interactions, something that would not be possible with AR HiChIP or ChIA-PET. Similar to published DNaseI hypersensitivity data [27, 36], our aggregated scATACseq showed that AR binding altered the chromatin accessibility (Fig. 5a). This led to an increased regional deviation between each ARBS enhancer class within DHT-treated cells (Fig. 5b). When comparing the relative fold change in accessibility, the largest increase was seen at inducible enhancers though both inactive and constitutive ARBS were significantly altered (Fig. 5c). As expected, AR binding led to a significant increase in the co-accessibility between multiple proximal ARBS following activation (Fig. 5d). When we incorporated these results into gene-specific networks, we observed that gained inducible enhancers frequently increased the number and complexity of interactions between ARBS. A representation of this alteration in co-accessibility is shown in Fig. 5e. To quantify these changes, we used our *cis*-regulatory networks to determine the relative impact of each AR CRE class on network complexity. In agreement with our proposed model, we found that inducible enhancers had the most significant impact on AR CRE interaction complexity and increased interactions with other ARBS (*p* < 4 × 10^−4^; Fig. 5f). Overall, inducible enhancers significantly increase network interactions between inactive and constitutive CREs and potentially act as a regulatory hub between promoters and other ARBS.

### Characterization of genetic alterations

**Fig. 6:**
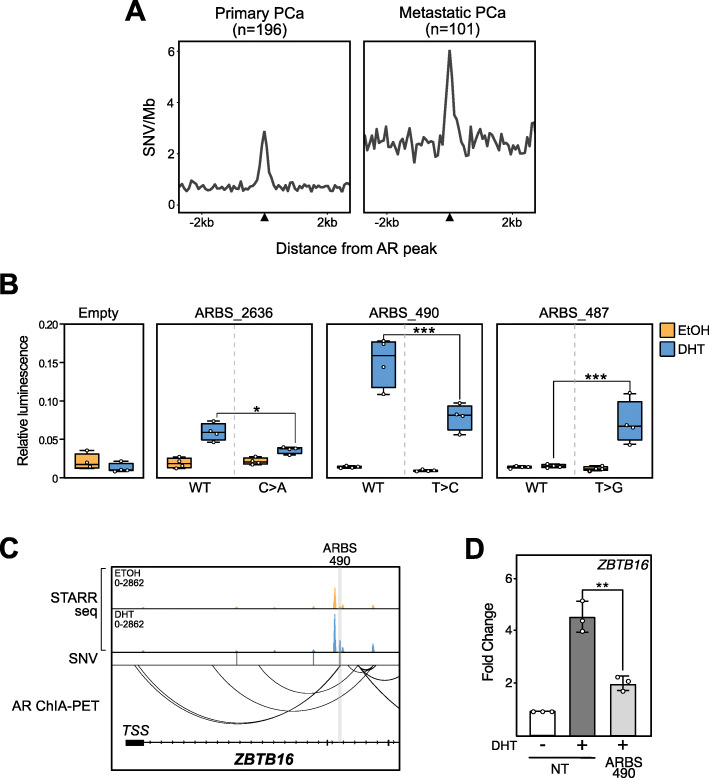
SNVs impact AR enhancer activity. **a** An increase in SNVs at ARBS is observed in both primary (left) and metastatic (right) PCa. **b** The impact of clinical SNV on androgen-dependent enhancer activity was quantified with a luciferase assay at inducible ARBS. Those SNVs that significantly altered AR enhancer activity (3/16) are shown (4 biological replicates ± SEM; *p < 0.05, ***p < 0.001). **c** Genome browser snapshot of *ZBTB16* gene locus. Gene looping is observed between enhancer ARBS_490 and *ZBTB16* promoter. **d** Expression of *ZBTB16* was quantified by qPCR after CRISPRi inhibition of ARBS_490. Androgen-induced expression of *ZBTB16* is suppressed compared to non-target (NT) gRNA control (3 biological replicates ± SEM; **p < 0.01)

Despite increasing evidence of non-coding driver mutations in PCa, these have been extremely difficult to identify due to both the poor annotation of CREs and the large number of non-coding somatic mutations. Given the critical function of inducible enhancers on AR-mediated gene expression, we speculated that non-coding mutations at these CREs could potentially alter gene transcription in PCa. To identify mutations at AR enhancers, we overlaid whole genome sequencing of primary PCa (*n* = 196) [37] and metastatic CRPC (*n* = 101) [19] with our functional enhancer annotations. As previously published, we observed a significant enrichment of single nucleotide variants (SNV) at ARBS in both primary PCa [22, 23] and also metastatic CRPC (Fig. 6a). Within the annotated ARBS, we found 751 SNVs in primary PCa and 1013 SNVs in metastatic PCa with 14% of the regions containing overlapping mutations (Additional file 1: Fig. S12A). Similar to most protein coding driver mutations in PCa, there were very few recurrent somatic mutations at ARBS [38]. We did not observe any difference in the SNV distribution between inducible, inactive, or constitutively active ARBS (two-sample Kolmogorov-Smirnov test, *p* > 0.5). To test the impact of these somatic mutations on AR enhancer activity, we focused on those metastatic SNVs that occur in inducible enhancers which looped to the TSS of AR-upregulated genes. Within the small set tested, we found that 19% (3/16) of SNVs significantly altered AR enhancer activity (Fig. 6b, Additional file 1: Fig. S12B). Interestingly, one of the affected enhancers was found to interact with the TSS of *ZBTB16*, a well-known AR-regulated tumor suppressor that is commonly mutated in CRPC [39, 40] (Fig. 6c). When this specific AR enhancer was inactivated with CRISPRi, we observed a significant decrease in the expression of *ZBTB16* (Fig. 6d). Taken together, these results show that non-coding SNVs at ARBS can impact enhancer activity of regulatory regions required for the AR-mediated gene expression.

## Discussion

The AR, like most nuclear receptors, binds to thousands of chromosomal sites but only regulates hundreds of genes [24, 41, 42]. The majority of AR-regulated gene promoters therefore physically interact with multiple ARBS [7]. Yet, how these binding sites work together to induce transcription is poorly understood, as annotation of non-coding CREs has been challenging. While descriptive approaches, including histone ChIPseq or GROseq, largely correlate with enhancer activity, they cannot provide the locus-specific resolution that is needed to understand these complex interactions. To annotate these individual regions, we systematically tested the enhancer activity of all commonly occurring clinical ARBS using an optimized STARRseq [26]. From this, we found that only a fraction of ARBS (7%) showed androgen-dependent enhancer activity, while the majority (81%) were inactive. This is analogous to work in stem cells where only a small percentage of CREs marked with NANOG, OCT4, H3K27ac, and H3K4me1 were found to be active enhancers [43, 44]. However, as we tested only a single time point, in an effort to limit the impact of androgen-induced DEG on AR activity, it is possible that there may be differing activity at ARBS at later time points. Yet, the results from this plasmid-based assay strongly correlate with epigenetic features in both PCa cell lines and clinical tumors, suggesting that the enhancer capability of the individual CRE is predictive of steady-state AR enhancer activity in situ (Figs. 1e and 2b). In matched patients pre- and post-ENZA treatment, we observed that H3K27ac at inactive and constitutive enhancers was generally not affected by AR inhibition, while inducible enhancers had a marked decrease following ENZA treatment. Supporting these results, in a larger patient population of primary PCa, H3K27ac was significantly enriched at both induced and constitutively active enhancers as compared to inactive ARBS (Fig. 2a). However, these descriptive ChIPseq results only broadly correlate with AR enhancer activity (Fig. 1g). They cannot annotate individual CREs and provide the resolution needed to characterize complex protein interactions, identify non-coding mutations, or delineate the underlying regulatory logic of AR-mediated transcription. By systematically testing all clinical ARBS for enhancer activity, this provides a detailed “map” needed to investigate these clinically important problems.

Using this functional annotation, we then characterized what features correlate with active enhancers. We initially interrogated DNA motifs in each AR enhancer class but this provided almost no predictive power as ARE motifs were equally distributed between inducible, inactive, and constitutive enhancers (Additional file 1: Fig. S7). This is in contrast to work with glucocorticoid receptors that suggested steroid response elements were more likely to be found at active enhancers [24]. Potentially, this may be due to the larger size of the ARBS tested, as many motifs will be randomly found in the large fragments (> 500 bp) used into our STARRseq library. Yet, while DNA motifs did not stratify enhancer CREs, many genomic features correlated with androgen-mediated enhancer activity. We expanded our analysis and trained a machine learning classifier to predict enhancer classes from all publicly available ChIPseq studies in LNCaP. Using this model, we could robustly predict inducible enhancers with a recall rate 10× greater than random ARBS. Importantly, we experimentally validated these predicted annotations and found that our model could successfully identify those regions likely to be inducible AR enhancers at a similar rate to our test set (Fig. 3b). Using this classifier, we identified those features that were predictive of AR enhancer activity (Fig. 3a). From this, we found that inducible enhancers strongly associate with both AR enhancer-associated features including H3K27ac and MED1, and co-activators such as PIAS1 and ARID1A [6, 45]. As this classifier is predictive for androgen inducible enhancers, it cannot identify those features which are critical, but broadly found, at many ARBS such as FOXA1. When each feature of this larger model was systematically down-sampled, we found that AR peak height could identify inducible enhancers at ARBS better, though not as well as our complete model, than any individual feature including H3K27ac (Fig. 3c). However, enhancer activity is not solely due to AR binding, as many inactive ARBS (196/2479) have a greater AR peak height than the median inducible enhancers.

Having annotated the different AR enhancers, we then characterized how each class impacted gene transcription. We found that inducible enhancers were more likely than inactive or constitutive enhancers to interact with either other ARBS or TSS of androgen-upregulated genes (Fig. 4a, b, e). Further, inducible enhancers had the highest number of chromosomal contacts of all ARBS and were central in AR CRE interaction networks (Fig. 4c, f). To test their role in transcription, we functionally inactivated each class of AR enhancers (Fig. 4g). Supporting their central role, all tested inducible enhancers strongly contributed to AR-mediated transcription. Surprisingly, transcription was not solely dependent on inducible enhancers and we observed that inhibition of specific constitutive or inactive ARBS also reduced androgen-induced transcription. This is unlikely to be an artifact of the STARRseq assay, as the annotations strongly correlate with enhancer features both in vitro and in vivo*.* While we cannot discount that these regions have enhancer activity in a different cellular context or time point, our results suggest that multiple ARBS CREs work in concert to drive gene transcription. In support of this model, we observed that those inducible enhancers interacting with additional ARBS have increased androgen-induced gene expression than those that do not (Fig. 4h). Further, we found that inducible, inactive, and constitutive AR enhancers had similar evolutionary conservation (Fig. 4i). It is unlikely that inactive or constitutive enhancers would be conserved, given the high rates of genetic drift in the non-coding genome, if they did not have a critical function in gene transcription. This proposed model of activity is not unique to AR and a similar phenotype has been observed with several other transcription factors. Recent work using CRISPRi to knockout individual estrogen receptor binding sites demonstrated hierarchical or synergistic interactions between enhancers on gene expression [46]. In addition, work with the antigen receptor showed that both active and inactive enhancers are required for gene expression [47]. Intriguingly, this suggests that enhancer activity is not the only feature required for AR-mediated transcription. While speculative, these inactive ARBS may work in concert with induced enhancers to increase the local protein concentration and potentially drive the formation of biological phase condensates that have been observed with other nuclear receptors at strongly active enhancers [48]. Supporting this model, we observed that AR binding at inducible enhancers significantly increased co-accessibility between ARBS (Fig. 5f) which causes inducible AR enhancers to be in close physical proximity to multiple ARBS (Fig. 4c). How these interactions occur is poorly understood and there is conflicting evidence that transcription factor binding can induce new chromatin loops or stabilize established interactions [34, 49]. Regardless of the mechanism, it is clear that inducible enhancers work with other AR CREs to drive AR-mediated gene transcription.

We and others have previously demonstrated that ARBS are highly mutated in primary PCa [22, 23]. Given the critical role of AR in PCa, we speculated that these somatic mutations could potentially alter the transcriptional activity of the tumor and potentially impact PCa growth and proliferation. However, selecting non-coding mutations for experimental validation is challenging due to both the large number of ARBS and SNVs and the relatively low frequency of recurrent mutations at CRE [50]. Further compounding this problem, mutations at multiple CRE can also impact gene expression thereby causing the same phenotype with different mutations [51]. Yet, while challenging to identify, non-coding somatic mutations can play a critical role in PCa disease progression [19–21]. By using our functional enhancer annotation to “map” specific CRE that are likely to impact androgen-mediated transcription, we stratified potential somatic mutations for testing. Focusing on inducible AR enhancers, we demonstrated that 19% of the SNVs tested significantly altered enhancer activity (3/16; Fig. 6b). This is significantly higher than a recent study using an orthogonal MPRA of H3K27ac sites which showed 1.8% of primary PCa SNVs impacting enhancer activity [23]. This difference could be due to technical issues related to insert size (146 vs. > 500 bp), the specific genomic regions tested (H3K27ac vs. inducible ARBS), the stage of PCa progression (primary vs. mCRPC), and the relatively small number of genomic regions tested (*n* = 16). Of particular interest, we identified a somatic mutation in metastatic PCa that significantly reduced the activity of a critical AR enhancer required for the expression of *ZBTB16* (Fig. 6d). This androgen-regulated gene is a well-characterized tumor suppressor that is frequently mutated in late-stage PCa [39, 52]. Immunohistochemical staining showed a significant reduction of *ZBTB16* in high-grade localized PCa specimens and weak or no expression in metastatic PCa biopsies [53]. *ZBTB16* knock-down experiments demonstrated increased PCa growth [39] and ectopic expression inhibited prostate cancer tumorigenesis in mouse models [54]. Further, loss of *ZBTB16* promotes a metastatic and ENZA-resistant phenotype in prostate cancer cells [39]. Given such a repressive function, homozygous *ZBTB16* mutations have been found to occur in 4–9% of CPRC tumors [40, 55, 56]. While these mutations occur in protein coding regions, our study demonstrates that somatic mutations in AR enhancer can also reduce *ZBTB16* expression.

In conclusion, we have created the first functional map of potential AR enhancer activity. By quantifying the activity of each ARBS, this provides mechanistic insight into mammalian gene regulation. Together, our data demonstrates that AR-driven inducible enhancers act as a regulatory hub that frequently cooperates with other ARBS to drive transcription. Identification of key enhancers can be used to stratify non-coding mutations for functional testing. These results have been integrated into an easy-to-use web app that is searchable by gene or genomic coordinates (https://lacklab.shinyapps.io/LSSHL/).

## Material and methods

### Cell lines

Cell lines were purchased from ATCC and routinely tested for mycoplasma contamination. LNCaP cells were routinely grown in RPMI 1640 media (Gibco) with 1% penicillin-streptomycin and 10% fetal bovine serum (FBS). No activation of the IFNγ pathway by double-stranded DNA was observed in electroporated LNCaP cells which have been shown to systematically alter STARRseq activity [26] (Additional file 1: Fig. S13A).

### Generation of ARBS STARRseq library

Common clinical ARBS were defined as those sites that were present in all normal prostate (*n* = 3) or independent PCa tumors (*n* = 13) [2]. Pooled human male genomic DNA (Promega) was randomly sheared (500–800 bp) using a Covaris M220 Focused-ultrasonicator. The fragments were end-repaired and ligated with Illumina compatible adaptors using the NEBNext Ultra™ II DNA Library Prep Kit (NEB). The adaptor-ligated DNA was hybridized to custom Agilent biotinylated oligonucleotide probes across a 700-bp region (53,032 probes; 4.684 Mbp oligo) and then pulled down by Dynabeads M-270 Streptavidin beads (NEB). The post-capture DNA library was amplified with STARR_in-fusion_F and STARR_in-fusion_R primers (Additional file 3: Table S1) and then cloned into AgeI-HF (NEB) and SalI-HF (NEB) digested hSTARR-ORI plasmid (Addgene plasmid #99296) with NEBuilder HiFi DNA Assembly Master Mix (NEB). The ARBS STARRseq library was transformed into MegaX DH10B T1R electrocompetent cells (Invitrogen). Plasmid DNA was extracted using the Qiagen Plasmid Maxi Kit.

### ARBS STARRseq

LNCaP cells (> 1.3 × 10^8^ cells/replica; 3 biological replicas) were electroporated with 266–300 μg (1 million cells:2 μg DNA) of the ARBS STARRseq capture library using the Neon Transfection System (Invitrogen). Electroporated cells were immediately recovered in RPMI 1640 medium supplemented with 10% FBS. After overnight recovery, the media were changed to RPMI 1640 medium supplemented with 5% charcoal-stripped serum (CSS) and 1% penicillin-streptomycin. Approximately 72 h after electroporation, the cells were treated with 10 nM DHT or EtOH for 4 h, washed with PBS, and then lyzed using the Precellys CKMix Tissue Homogenizing Kit and the Precellys 24 Tissue/Cell Ruptor (Bertin Technologies). Total RNA was extracted using Qiagen RNeasy Maxi Kit (Qiagen), and the mRNA was isolated using the Oligo (dT)_25_ Dynabeads (Thermo Fisher). Isolated mRNA samples were treated with Turbo DNase I (Thermo Fisher), synthesized into cDNA using the gene-specific primer (Additional file 3: Table S1), treated with RNaseA (Thermo Fisher), and PCR amplified (15 cycles) with the junction PCR primers (RNA_jPCR_f and jPCR_r primers, Additional file 3: Table S1). The ARBS STARRseq capture library was PCR amplified with DNA-specific junction PCR primers (DNA_jPCR_f and jPCR_r primers, Additional file 3: Table S1). After junction PCR and AMPure XP beads clean-up, an Illumina compatible library was generated by PCR amplification with TruSeq dual indexing primers (Illumina) and sequenced on Illumina HiSeq4000 (150 bp; PE). The resulting sequencing data is available at GSE151064.

### Analysis of STARRseq data

Reads were mapped to reference genome (hg19) with BWA aligner (v0.7.17) [57] and all mapped reads with a MAPQ score < 60 or indels were removed. Captured region coverage was quantified with BamCoverage function (v3.1.3) in DeepTools [58] while discarding all reads on blacklisted regions (ENCODE ENCFF001TDO). Differential enhancer activity was quantified by DESeq2 (v1.26.0) [59]. Induced enhancers were defined as having a log2 fold change (LFC) > 1 and *p*-adj < 0.05 in the DHT/EtOH samples. Constitutively active enhancers had plasmid-normalized reads LFC > 1 in both EtOH and DHT but no DHT induction (DHT/ETOH LFC < 1). Inactive regions had both minimal DHT inducible activity and low plasmid to RNA ratios (plasmid-normalized LFC < 1). Output of the DESeq2 was visualized with ggplot2 [60].

### Clinical approval and sample collection

Clinical PCa tissue was collected before and after enzalutamide (ENZA) therapy from the Dynamics of androgen receptor genomics and transcriptomics after neoadjuvant androgen ablation study (ClinicalTrials.gov #NCT03297385). The trial was approved by the IRB of the Netherlands Cancer Institute. Informed consent was signed by all participants enrolled in the study, and all research was carried out in accordance with relevant guidelines and regulations. Trial participants received 3 months of neoadjuvant ENZA treatment prior to robotic-assisted laparoscopic prostatectomy. Biopsy (pre-treatment) and prostatectomy specimens (post-treatment) were fresh-frozen and sectioned prior to immunoprecipitation. Tissue sections were examined pathologically for tumor cell content, and only those samples with a tumor cell percentage of ≥50% were used for tissue ChIPseq analysis.

### Tissue ChIPseq

ChIP on prostate cancer biopsy and prostatectomy tissues were performed as previously described [61]. Nuclear lysates of each tissue specimen were incubated with 5 μg of H3K27ac antibody (Active Motif, 39133) pre-bound to 50 μL magnetic protein A Dynabeads (Thermo Fisher Scientific, 10008D). Immunoprecipitated DNA was processed for library preparation (Part# 0801-0303, KAPA biosystems kit), and samples were sequenced using an Illumina HiSeq 2500 system (65 bp, single-end). Sequences were aligned to the human reference genome hg19, duplicate reads were removed, and reads were filtered based on MAPQ quality (≥ 20).

### Tissue ChIPseq data processing

Intensity plots were generated using EaSeq [62]. For boxplots, the number of sequence reads per region of interest was calculated using bedtools multicov (v2.25.0) [63]. The data was further processed in R (v3.4.4). Region read counts were *z*-transformed per sample to correct for differences in total read count. Statistical significance in read counts differences was determined using the Mann-Whitney test, based on the median read count over all samples, and adjusted for multiple testing using FDR.

### RNAseq and ChIA-PET analysis

AR-regulated genes were identified from publicly available RNAseq data of LNCaP cells treated with 100 nM DHT for 6 h (GSE64529) with DESeq2 (v1.26.0) [59]. The distance between androgen-upregulated genes and ARBS was calculated with HOMER (v4.10) [64]. The resulting data was merged in 100-bp bins and the cumulative distribution function was determined with *Ecdf* in R (v3.6.1). Specific ARBS-promoter interactions were identified from the published AR ChIA-PET performed in VCaP cell line (GSE54946) by overlapping the loop end of either ARBS or gene’s TSS (± 5Kb) with GenomicInteractions R package (v1.20.3) [65].

### ChIPseq and GROseq analysis

Previously published ChIPseq and GROseq data were downloaded from the GEO database and uniformly processed (Additional file 3: Table S1). The sequencing reads were controlled for quality using FASTQC, and the reads were then mapped to the human reference genome (hg19) with bowtie aligner (v0.12.9) [66]. All reads mapped to the blacklisted regions (ENCODE ENCFF001TDO) were discarded. For direct comparison of the H3K27ac ChIPseq with the STARRseq data, average signal values of the inducible and inactive regions were calculated using bigWigAverageOverBed software(v377) [67]. For each region, log fold change values were calculated between DHT and ETOH treatments in H3K27ac and STARRseq experiments. Scatterplots were generated by ggplot2 R package (v3.2.0) [60]. ROSE [68] was used to identify super enhancers from DHT H3K27ac ChIPseq. BigWig signal tracks were generated with BamCoverage function (v3.1.3) of deepTools [58] with RPKM normalization. For GROseq data, using log fold enrichment between + strand of DHT over the + strand EtOH, a single track was generated. Similarly, a single − track was also generated. Then − strand is subtracted from + strand to final bigwig file. For each of the 500 positive control enhancer regions, we calculated the average accessibility scores using bigWigAverageOverBed from ENCODE’s LNCaP DHT (ENCFF975MZT) and EtOH (ENCFF906QXX) DNase-seq experiments. Then we compared the top and bottom 100 to show differences in accessibility. Later, for the same regions, we calculated the average STARRseq signal with bigWigAverageOverBed [67] from merged DHT and EtOH samples.

### Conservation

One hundred vertebrate species conservation track was obtained from UCSC golden path (http://hgdownload.cse.ucsc.edu/goldenpath/hg19/phastCons100way/hg19.100way.phastCons.bw) for hg19 reference. The average conservation distribution of induced, constitutive, inactive ARBS enhancers and negative control regions were calculated with deeptools’ *computeMatrix* function (v3.1.3). Output distribution matrix then visualized with deeptool’s *plotProfile* function (v3.1.3).

### Network graph of AR CRE

A network graph was built using the annotated CREs (clinical ARBS and TSS) and LNCaP ARBS (not tested for enhancer activity) as nodes and the chromosomal interactions from VCaP ChIA-PET as edges. The interactions between each enhancer class or ARBS were extracted from processed data and introduced as a graph with NetworkX (v2.3). Only TSS of AR DEG were considered in the network. The annotated CRE network graph contained 2361 nodes, 1951 edges, and 830 separate components. The annotated CRE and non-annotated ARBS graph contained 10,484 nodes, 12,437 edges, and 1873 separate components. The relative density (Eq. 1) of two different classes was calculated based on the expected frequency between two classes. If self-looping occurred within the same class, the expected maximum edge frequency was duly calculated to reduce duplication (Eq. 2). For different classes in the bipartite graph, the expected maximum edge frequency is calculated accordingly (Eq. 3). The interaction frequency (Eq. 4) was scored as the ratio of observed versus expected edge number relative to whole graph density.

1 $$ D=\frac{2{E}_{\mathrm{obs}}}{V\left(V+1\right)} $$

2 $$ {E}_{\mathrm{max}}={C}^R\left(V,2\right)=\frac{V\left(V+1\right)}{2} $$

3 $$ {E}_{\mathrm{max}}=V\times U $$

4 $$ {IF}_{\left(U,V\right)}=\frac{E_{\mathrm{obs},\left(U,V\right)}}{D\times {E}_{\mathrm{max}}} $$

Degree centrality and betweenness centrality scores were calculated by NetworkX. Although a not-connected graph gives information about the whole network, it is biased when comparing the centrality features of all elements. Therefore, we calculated the centrality score from the largest connected component.

### Enhancer luciferase assay

The region of interest (750–850 bp) was PCR amplified from pooled male human DNA (Promega) and cloned into a STARRseq luciferase validation vector_ORI_empty plasmid (Addgene plasmid #99298) with HiFi DNA builder (NEB). Primers used to amplify specific regions are described in Additional file 3: Table S1. LNCaP cells were co-transfected with 500 ng reporter plasmid and 5 ng of Renilla using TransIT-2020 (Mirus) and plated in phenol red-free RPMI (Gibco) supplemented with 5% CSS (Fisher Scientific). Forty-eight hours post-transfection, cells were treated with 10 nM of DHT or ETOH for 24 h. Firefly and Renilla luciferase activity were assayed by Dual-Glo Luciferase assay system (Promega). All the experiments had a minimum of 4 biological replicas with 3 technical replicates in each experiment. Single nucleotide substitutions of reporter constructs were carried out by inverse PCR mutagenesis as previously described [69]. All mutagenic primers are given in Additional file 3: Table S1.

### gRNA design and CRISPRi

Multiple gRNAs per enhancer region were designed with CRISPR-SURF [70] and cloned into lentiGUIDE-puro (Addgene #52963). All gRNA sequences are provided in Additional file 3: Table S1. LNCaP cells stably expressing dCas9-KRAB were generated by transducing LNCaP cells with Lenti-dCas9-KRAB (Addgene #89567) followed by blasticidin selection. dCas9-KRAB expression was confirmed by Western blot (Cas9: CST Mouse mAB #14697, GAPDH: SC Rabbit pAB #25778) (Additional file 1: Fig. S13B). LNCaP-dCas9-KRAB cells (200,000/well) were transfected with 1 μg of gRNA using Mirus TransIT-X2 and selected with puromycin (2 μg/ml) for 72 h. The media were then changed to phenol red-free RPMI supplemented with 5% CSS and treated with EtOH or 1 nM DHT for 24 h. Androgen-induced expression was quantified by qRT-PCR using gene-specific primers (Additional file 3: Table S1). As CRISPRi is known to be prone to false negatives, all gRNAs were initially tested and then a single gRNA was used against each genomic region. In each set, the expression of the non-AR-regulated gene *FXN* was also quantified to assess non-specific inhibition. Each experiment was done in triplicate with a minimum of 3 biological replicas.

### Single cell ATACseq (scATACseq)

LNCaP cells were cultured in phenol red-free RPMI 1640 media supplemented with 5% CSS and 1% antibiotics for 60 h and then treated with 100% EtOH or 10 nM DHT for 4 h. Nuclei from 1 × 10^6^ cells were isolated according to 10x Genomics recommended protocol (Nuclei Isolation for Single Cell ATAC Sequencing CG000169 Rev D). scATACseq libraries were prepared using 10x Genomics Chromium Next GEM Single Cell ATAC Library & Gel Bead Kit v1.1. Libraries were sequenced on a NextSeq500 Illumina sequencer (23,428 unique PE reads/cell DHT and 27,077 unique PE reads/cell EtOH). scATACseq analysis was done with 10X Cell Ranger software. Deviation/*z*-score was calculated with chromVAR [71] and UMAP was calculated in ArchR [72] (v0.9.3). ATACseq peaks were called using MACS (v2.2.6) [73]. Random genomic regions were generated using bedtools (v2.29.2) [74]. scATACseq co-accessibility scores in EtOH- and DHT-treated LNCaP scores were calculated using Cicero (v1.4.0) [35] with 10X Genomics input. Co-accessible sites overlapping with STARRseq annotated AR enhancers and LNCaP ARBS (GEO:GSM2219854) were calculated with bedtools (v2.29.2) [63] and counted with R (v3.6.1). With these results, two network graphs (EtOH+DHT) were built with annotated AR CREs, LNCaP ARBS (not present in STARRseq library) and promoters as nodes with the predicted Cicero interactions as edges (co-accessibility score > 0.1). Inexact graph matching was used to calculate the similarity score of these networks with the DeltaCon, a massive-graph similarity function algorithm [75]. The *S* affinity matrices were calculated as described (Eq. 5) where *I* is the identity matrix, *D* is the diagonal degree matrix, and *A* is the adjacency matrix of *G* and *ε* is the constant defined as $\varepsilon =\frac{1}{1+{\max}_i\left({d}_{ii}\right)}$. Next the Euclidean distance of two affinity matrices (*d*) was calculated (Eq. 6).

5 $$ S=\left[ sij\right]={\left[I+{\varepsilon}^2D-\varepsilon A\right]}^{-1} $$

6 $$ d= RootED\left({S}_1,{S}_2\right)=\sqrt{\sum_{i=1}^{n_i}{\sum}_{j=1}^{n_j}{\left(\sqrt{s_{1, ij}}-\sqrt{s_{2, ij}}\right)}^2} $$

If an edge adjacent to the node (*v*) was altered, the impact (*w*) was calculated as the *RootED* distance between corresponding row vector *S*_1, *v*_ and *S*_2, *v*_ calculated where *v* = *j* = 1 → *n*_*j*_ and *n*_*i*_ = 1. Otherwise, *w* = 0.

### Machine learning of ARBS activity

All ChIPseq data used in machine learning was analyzed with the standardized ChIP-Atlas bioinformatic pipeline [76]. Based on the inducible (*N* = 286), inactive (*N* = 2479), and constantly active (*N* = 465) categorization of the clinical ARBS, we trained a classifier to predict the groups using the bound factors in a given region as input. For each ARBS region, we extracted the ChIPseq signal scores over a 750-bp region for 90 different DNA binding factors (Additional file 2). To correct for variations in scores across factors and to unify their values to a consistent range, we applied SES normalization to estimate a score cutoff that separates non-specific from specific binding in each ChIPseq dataset [77]. Briefly, the method finds the score where the difference between the cumulative distributions of the observed and control input scores is maximally different. The median value of the scores above the binding cutoff was then used as the mean of a sigmoid transformation for each bound factor to transform individual factors to an occupancy score between 0 and 1. Heatmaps of the average occupancy score for each bound factor at a 50-bp resolution for inducible and inactive enhancers are shown in Additional file 1: Fig. S14A + B. Finally, we took the maximum occupancy score over the 750-bp region as the feature of the factor’s activity. The classifier we chose to fit was a bootstrapped multinomial logistic regression model with a sparsity LASSO regularizer. Several other regularizations were tried including Ridge and Elastic but this was found to give the best accuracy and interpretability. In an attempt to balance the number of samples between groups, we created dataset samples that consisted of 500 randomly selected samples of the Non-Inducible group alongside all of the samples from the Constitutively Active and Inducible ARBS groups. The data set was further split into 80% training and 20% testing. The bootstrapped model consists of 100 thousand different base logistic regression estimators which were all trained on a different subset of the training data. Each subset contains a maximum of 50 training samples and 5 occupancy features. The fitted weights in the aggregate model are an average of the fitted weights in the base estimators and reflect the importance of a factor to differentiate the categories in the presence and absence of other factors. This eliminates the problems of collinearity and the linear dependence within our features and allows for a robust representation of feature importance. Assessing these classifiers showed both false negatives (Additional file 1: Fig. S14C) and positives (Additional file 1: Fig. S14D). False negatives were likely due to missing values in the ChIPseq dataset as this group was much more likely to have a missing value than the inducible group as a whole. False positives were still mostly above zero, indicating potential enhancer function that fell just below the fold-change cutoff to be considered in the inducible group (Additional file 1: Fig. S14D). To rank the different factors in terms of predictive power for a given group, we computed the average binding energy of a given factor as the fitted weight for that group times the average occupancy for that factor over all regions in that group. The difference in binding energies between groups could then be used to identify features that differentially predict one group over another. For the 10,000 LNCaP-specific ARBS regions, we computed their occupancy and constructed their 90 input features as above. The trained classifier was then used to predict the probability of each of the three categories for each of these regions using their occupancy features as input.

## Supplementary Information

**Additional file 1: Fig.S1- S14.** Supplementary figures.

**Additional file 2.** All the publicly available data used.

**Additional file 3: Table S1.** Primers and gRNAs.

**Additional file 4.** Review history.

## Data Availability

All datasets generated during this study along with other processed files are available at NCBI’s Gene Expression Omnibus (GEO) under the accession GSE151064 [78]. All publicly available data used can be found in Additional file 2.
