# Supplementary material for: Functional mapping of androgen receptor enhancer activity
Source: Genome Biol. 2021 May 11;22:149. doi: 10.1186/s13059-021-02339-6 (PMC8112059; doi:10.1186/s13059-021-02339-6)
Supplement: Supplementary file 1 — Additional file 1: Fig.S1- S14. Supplementary figures. [file 13059_2021_2339_MOESM1_ESM.pdf]

Figure S1

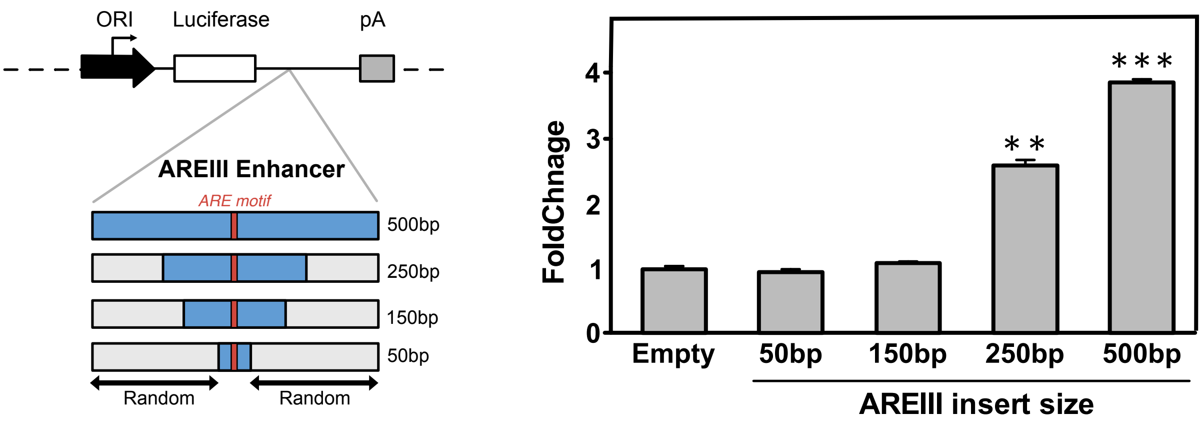

Figure S1: ARBS size is critical for its enhancer functions. Varying lengths of AREIII enhancer flanked with random sequences were cloned into the modified STARR ORI luciferase plasmid (left). The ARE motif which is centroid in location is highlighted in red. Androgen-activated activity of these size-matched constructs were measured by luciferase assay and the data is represented as the fold change (EtOH/DHT) normalized to empty plasmid (3 biological replicate±SD; ** $p < 0.01$). (Right)

Figure S2

A

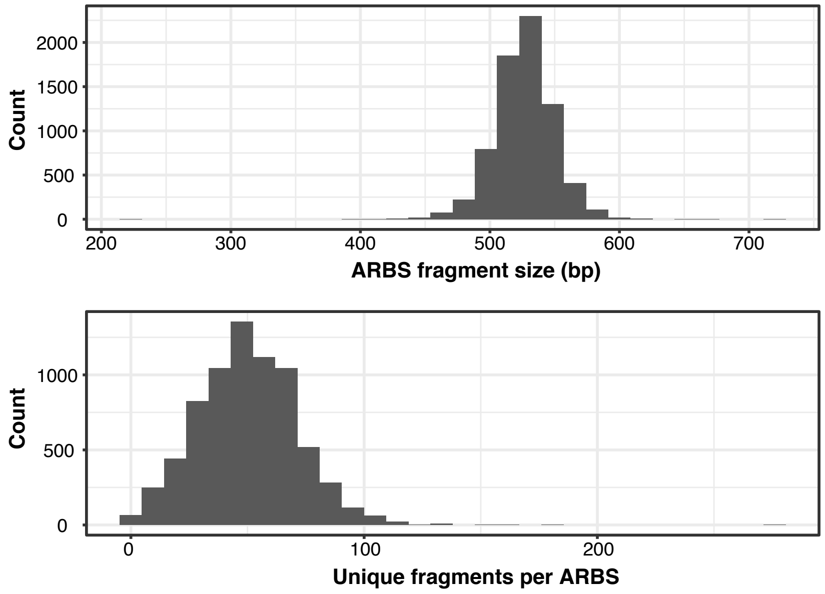

B

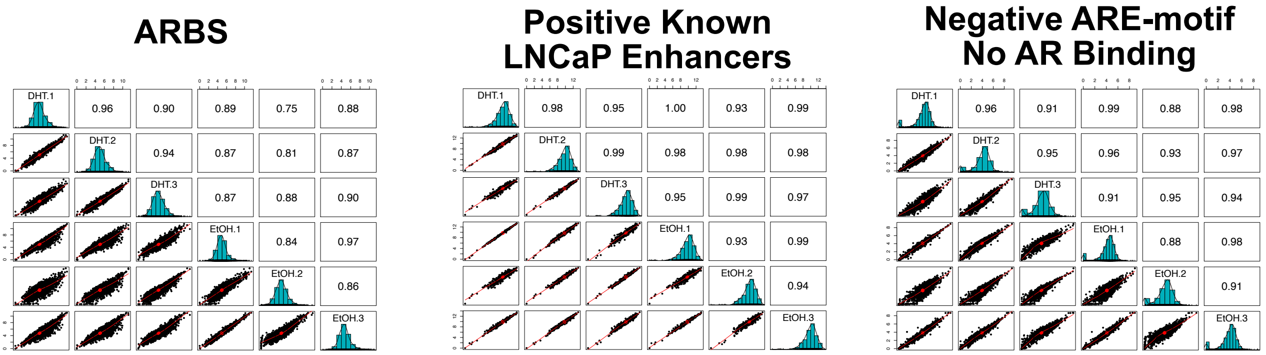

Figure S2: (A) Clinical ARBS were captured from normal genomic DNA using a custom-capture and cloned into STARRseq plasmid library. The capture STARR-seq library contains the ARBS with the median size of >500bps (top) and a normal distribution of the fragments covering the whole of the capture region (bottom). **(B)** The Pearson correlation of STARRseq self-transcription counts from each LNCaP biological replica (EtOH 1-3 and DHT1-3) was compared with tested regions in the plasmid library including clinical ARBS, known non-AR enhancers and sites that contain an ARE but not AR binding. We observed excellent correlation between all 3 biological replicates (Pearson correlation 0.84-0.99).

Figure S3

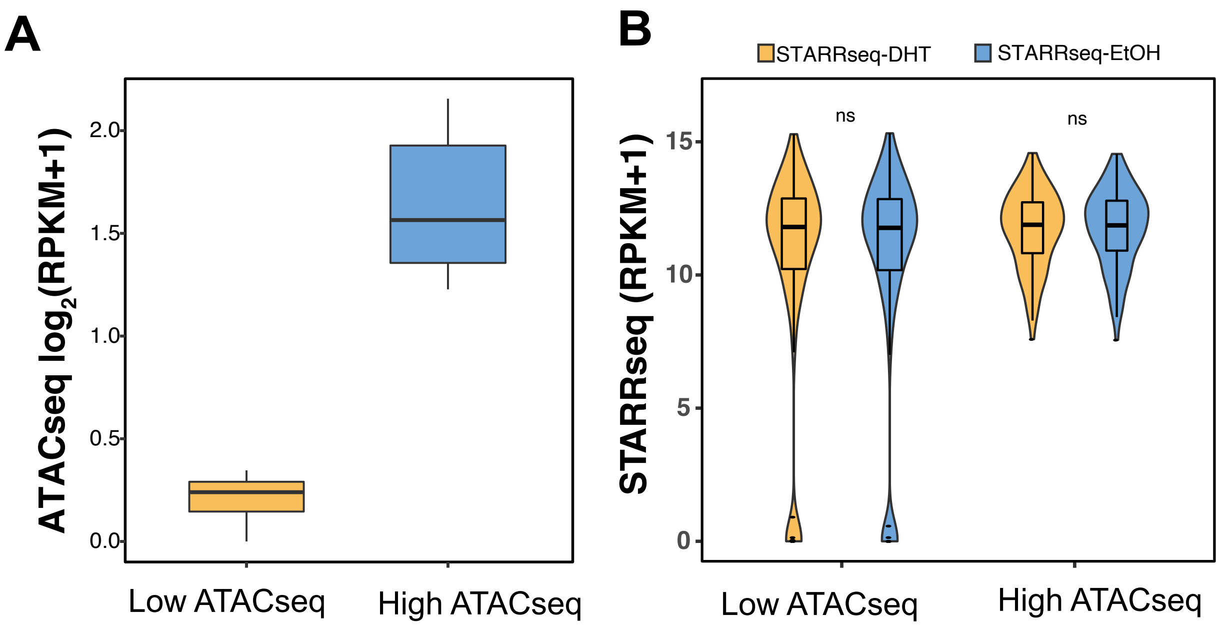

Figure S3: (A) Non-AR LNCaP enhancers (positive control) were subset into two groups based on their chromatin compaction in LNCaP cells (GSE148925); heterochromatin (n=100; Low ATACseq) or euchromatin (n=100; High ATACseq). **(B)** The violin plot shows the STARR-seq enhancer activity of these regions with low DHS and high DHS. Similar enhancer activity is observed in both the groups regardless of the endogenous chromatin compaction.

Figure S4

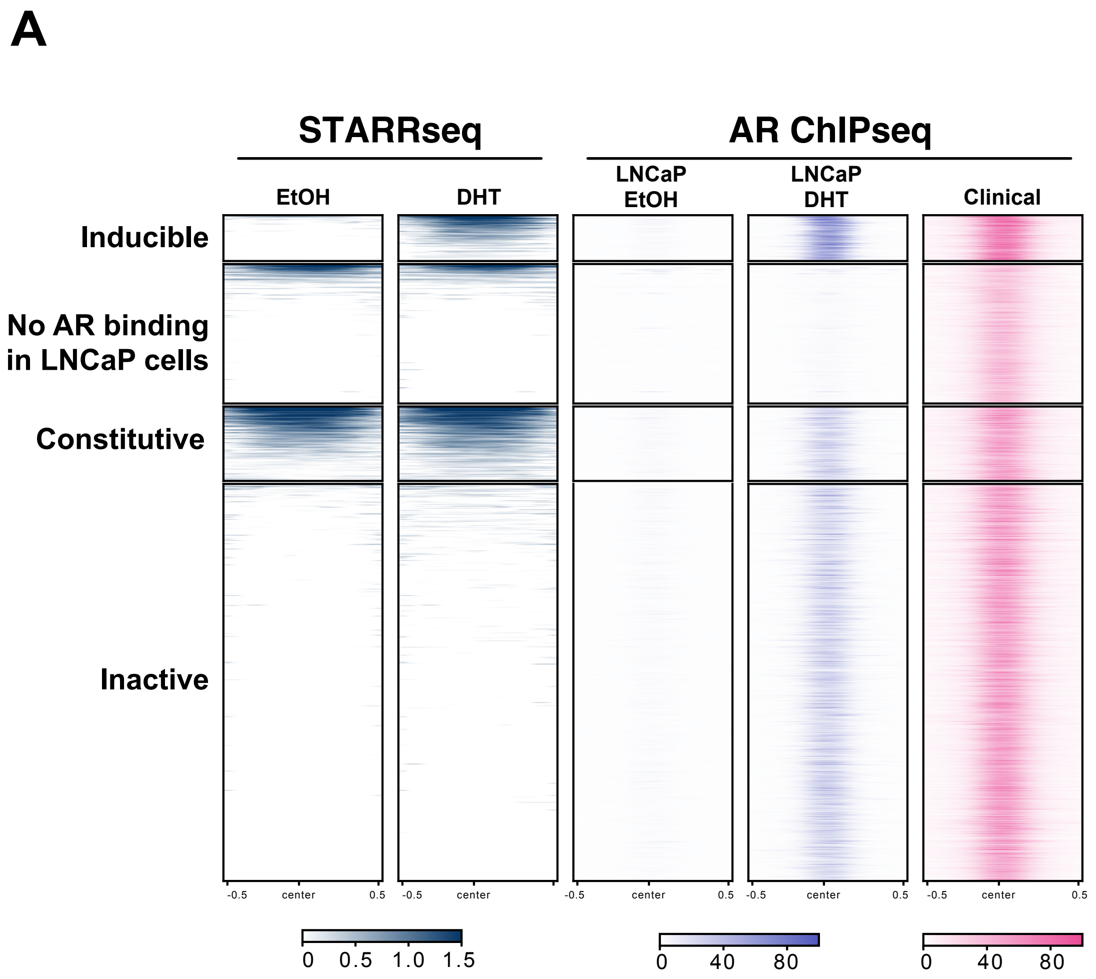

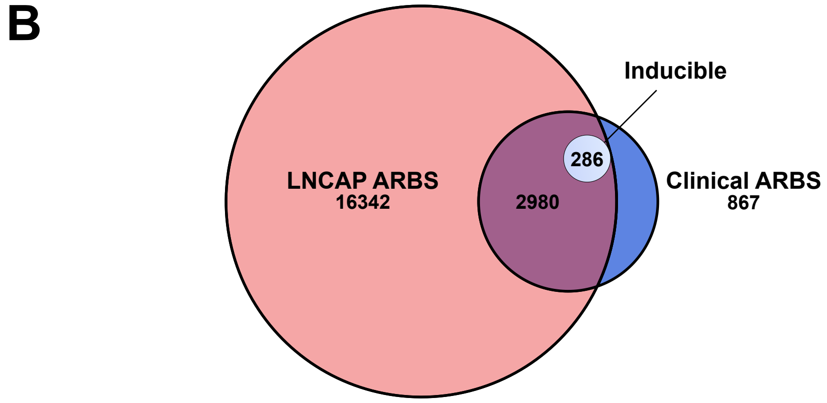

Figure S4: (A) Heatmap of STARR-seq and AR ChIPseq in LNCaP cells and primary PCa samples (n=13). ARBS found only in PCa clinical tissue specimen but not in LNCaP cells (n=867) termed as 'no AR' shows no enhancer activity in LNCaP. **(B)** Venn diagram shows the overlap of ARBS in clinical samples and LNCaP cells. Only those ARBS common to both clinical samples and LNCaP cells show androgen-dependent enhancer activity.

Figure S5

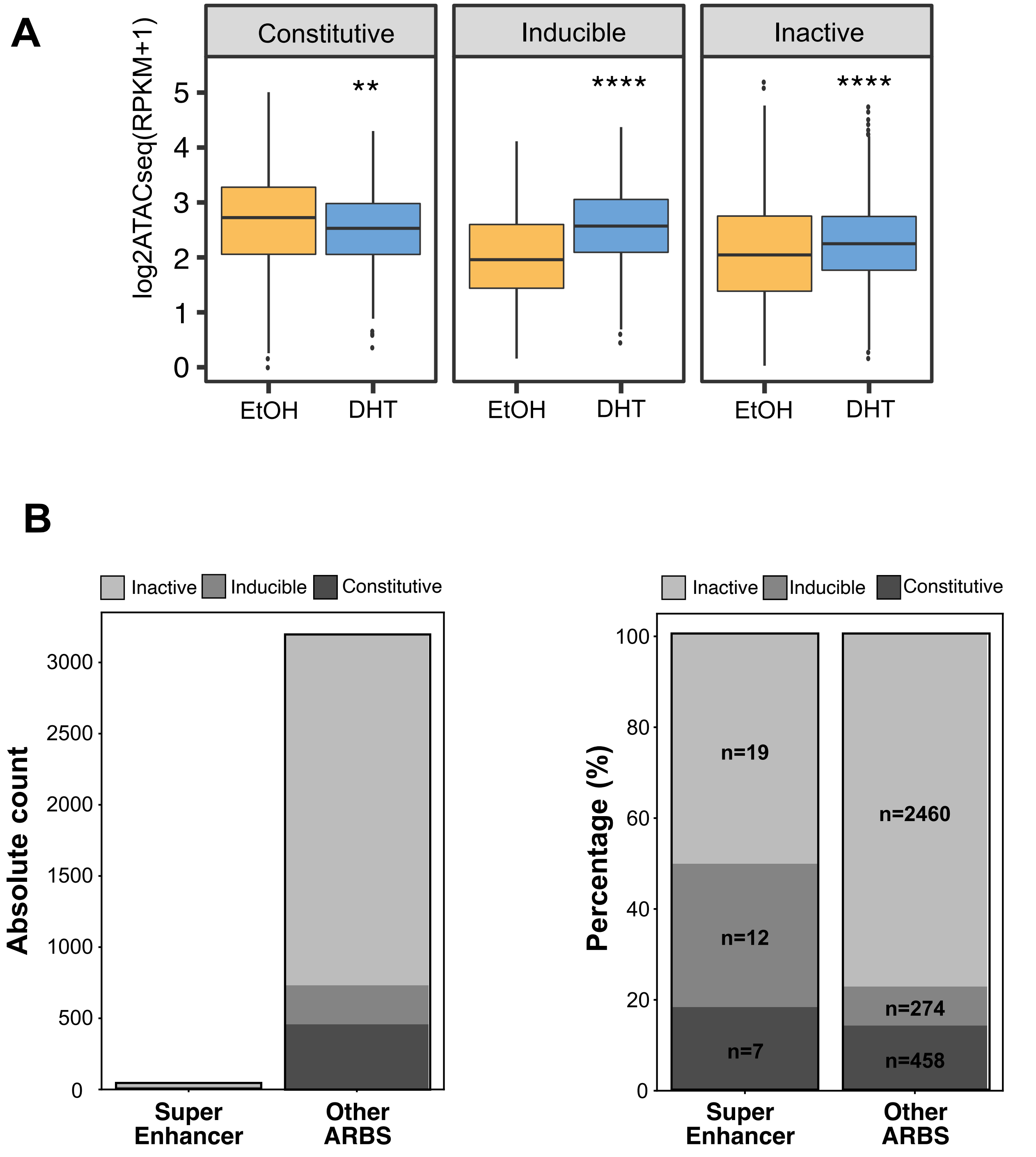

Figure S5: (A) Published ATACseq data from LNCaP cells treated with 4h of DHT/ ETOH (GSE148925) were analyzed to compare the differences in chromatin accessibility. A significant increase in chromatin accessibility is observed in all enhancer classes after DHT treatment (** p=1.6e-03, **** p<3.1e-013). **(B)** Super enhancers score of ARBS in each enhancer class were calculated by ROSE (Methods). Enrichment of super enhancers in absolute count (left) and relative enrichment (right) among different classes of enhancers are shown. No enrichment of super enhancers were observed in the ARBS tested by STARRseq.

Figure S6

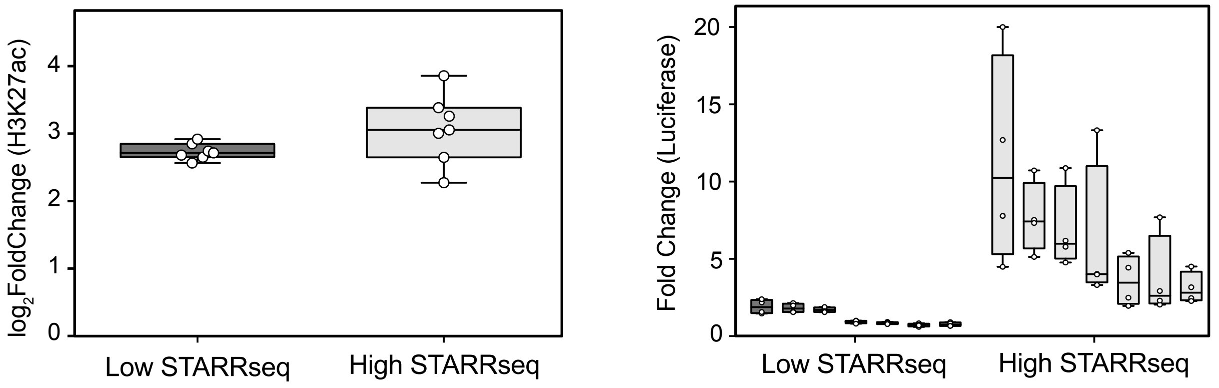

Figure S6: (Left) ARBS were divided into two groups; low-inducible (n=7) or highly-inducible (n=7) based in their STARR-seq signals.The box plot shows the log fold change (DHT/EtOH) of H3K27ac (GSE51621) signal at these regions. No statistically significant difference in the activity is observed. Androgen- dependent enhancer activity of these regions were further tested by conventional luciferase reporter assay. **(Right)** Results from the luciferase assay (4 biological replicates) of all the regions are shown. While both the groups showed high H3K27ac signal, only the inducible STARRseq group shows androgen-dependent reporter activity in the luciferase assay.

Figure S7

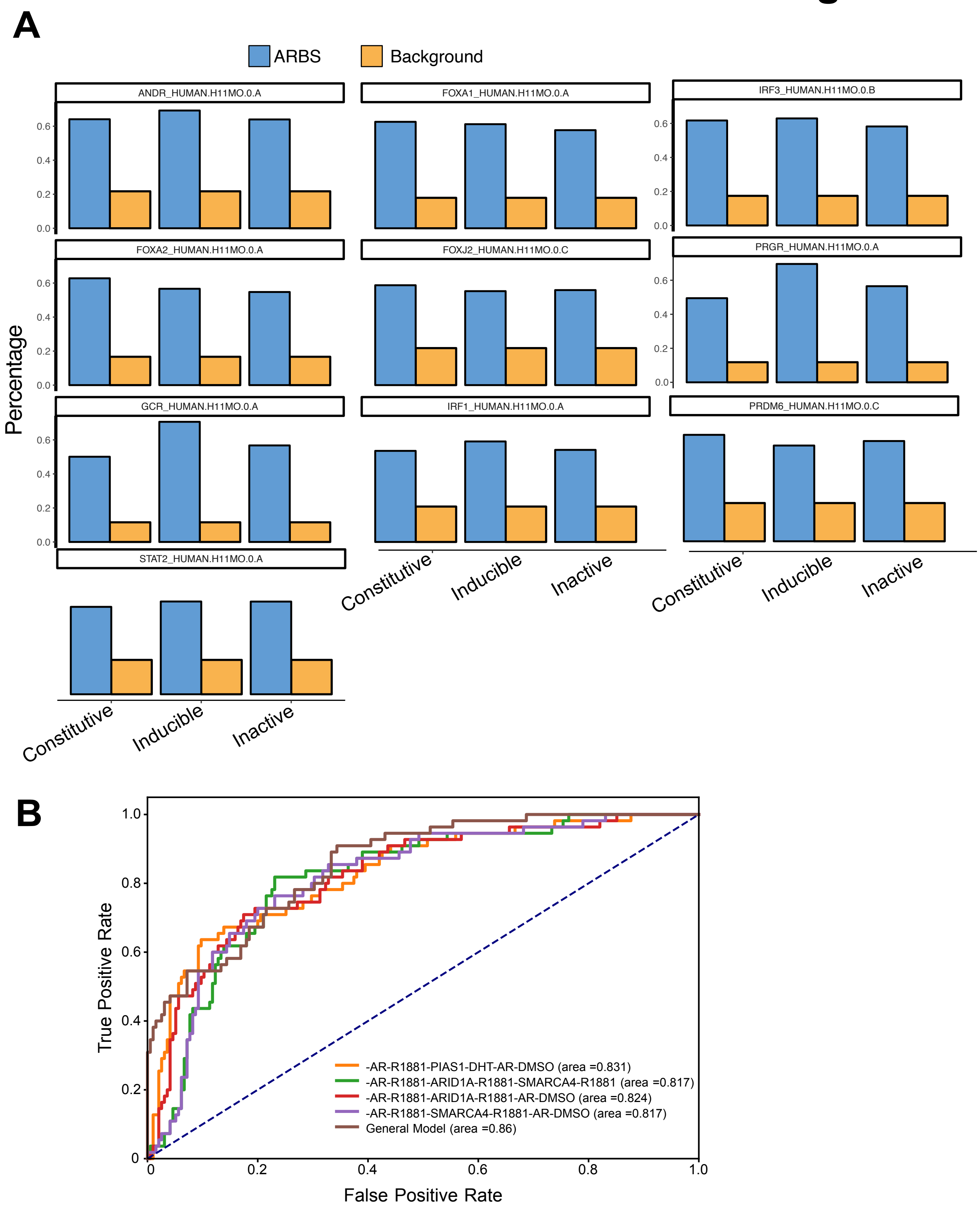

Figure S7: (A) Motif analysis was performed to identify the features associated with each enhancer class. No difference in motif enrichment was observed among different classes of enhancers. Enrichment of top (n=10) highly enriched motif in relation to the background in all 3 different classes of enhancers are shown. **(B)** Receiver operating characteristic curve of the top three downsampled features (4/117480 permutations) from the machine learning classifier compared to the larger general model.

Figure S8

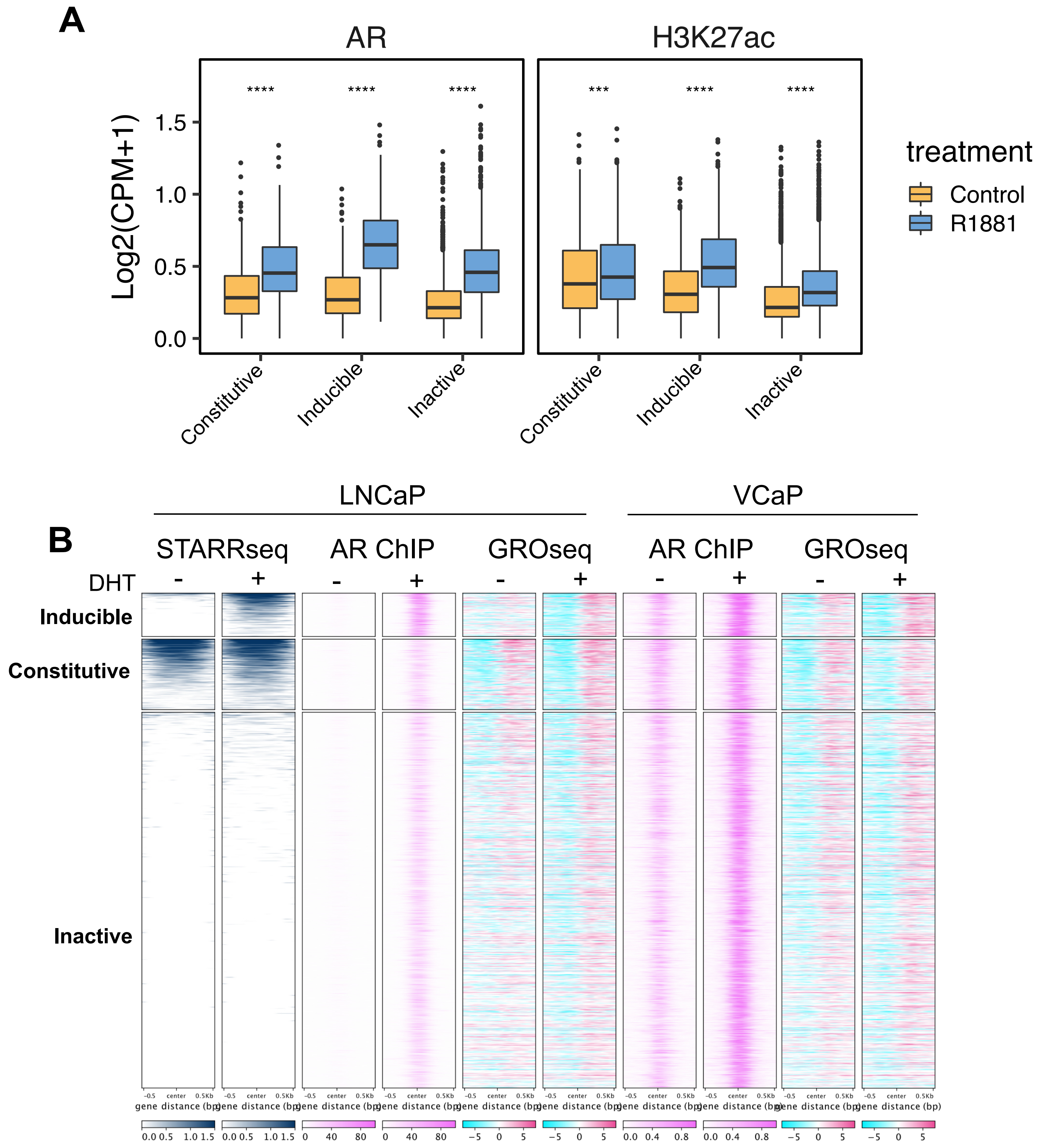

Figure S8: No difference in AR activity is observed in LNCaP and VCaP cells (A) Box plot shows the AR and H3K27ac binding to the different classes of enhancers in EtoH and DHT treated VCaP cells. (B) Heatmap shows STARRseq (Blue),AR ChIP (Pink) and Groseq with normalized read counts from positive (Cyan) and negetive (Pink) bidirectional RNA in both LNCaP and VCaP cells. The heatmap is divided based on different classes of enhancer identified by STARRseq

Figure S9

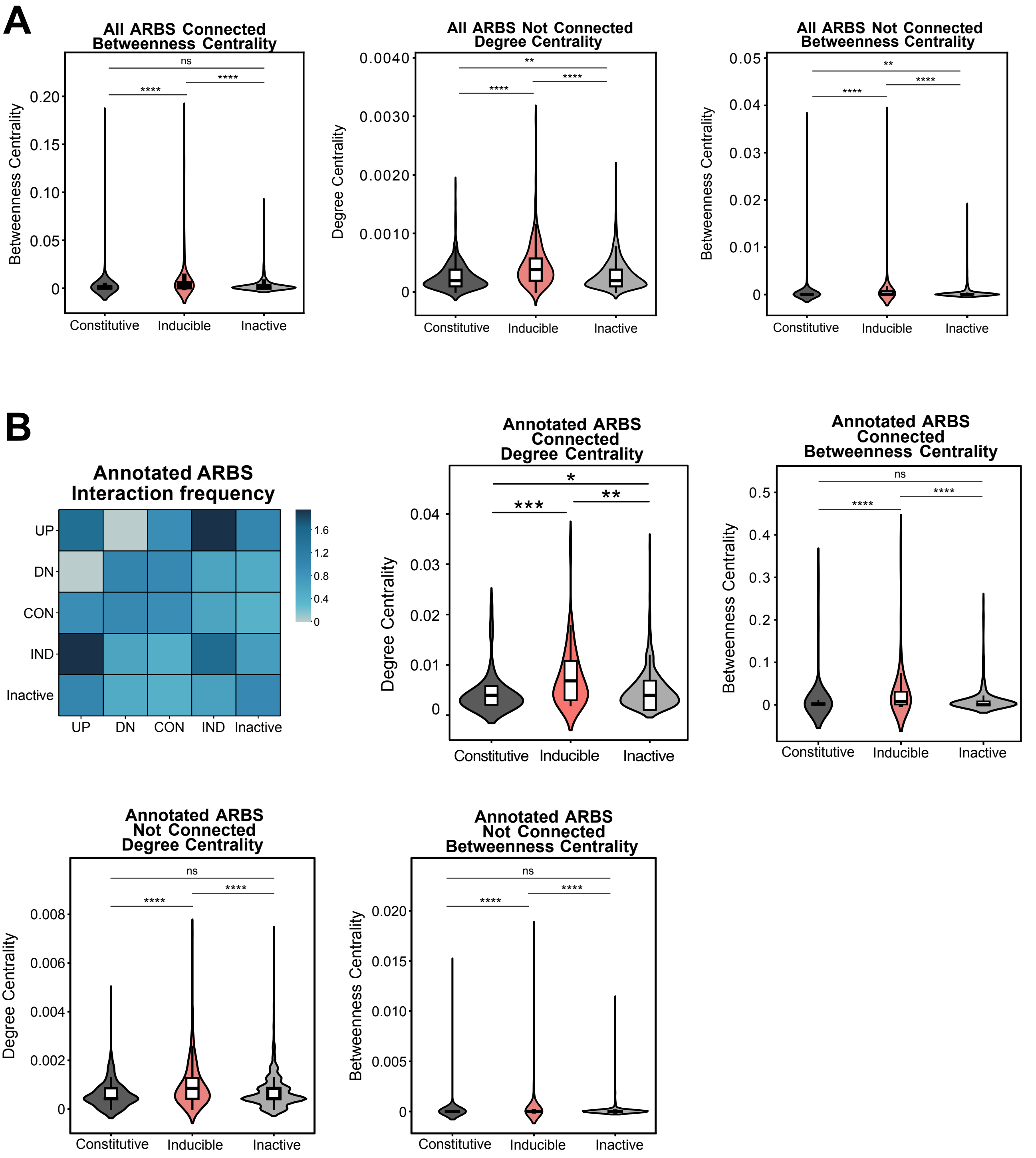

Figure S9: Interaction network of clinical ARBSs. **(A)** In the largest connected network the betweenness centrality score is shown for all AR enhancer classifications. **(B)** For the complete network the degree centrality score (left), and betweenness centrality scores (right) are shown for all AR enhancer classification. **(C)** In an interaction network of only annotated clinical ARBS the interaction frequency between each node class (top-left), degree centrality scores in the biggest connected component (top-middle), betweenness centrality scores in the biggest connected component (top-right), degree centrality scores for the whole network (bottom-left), and betweenness centrality score for the whole network (bottom-right). (ns *p>0.05, **** p<10-9*)

Figure S10

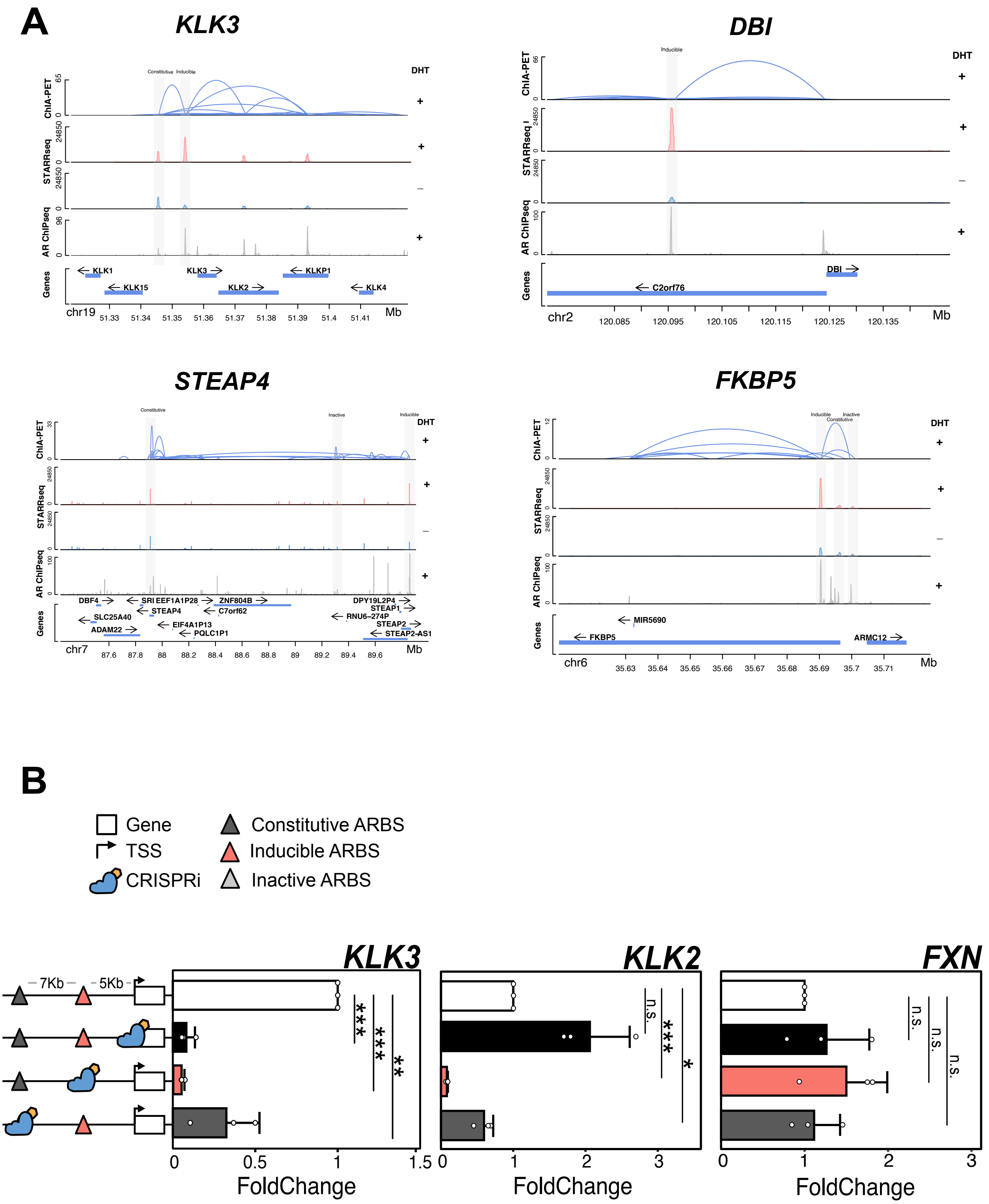

Figure S10: (A) Genome browser snapshot of cis-regulatory regions around characterized AR- regulated genes. Long-range chromatin interactions are shown in blue. STARR-seq peaks are shown in red and AR ChIP-seq peaks in grey. (B) The bar graph shows the DHT mediated activation of KLK3 and KLK2 quantified by qPCR following CRISPRi targeting either KLK3 promoter or KLK3 enhancers. FXN is the off-target control (3 biological replica± SD, *** p<0.001, ** p<0.01 and * p<0.05).

Figure S11

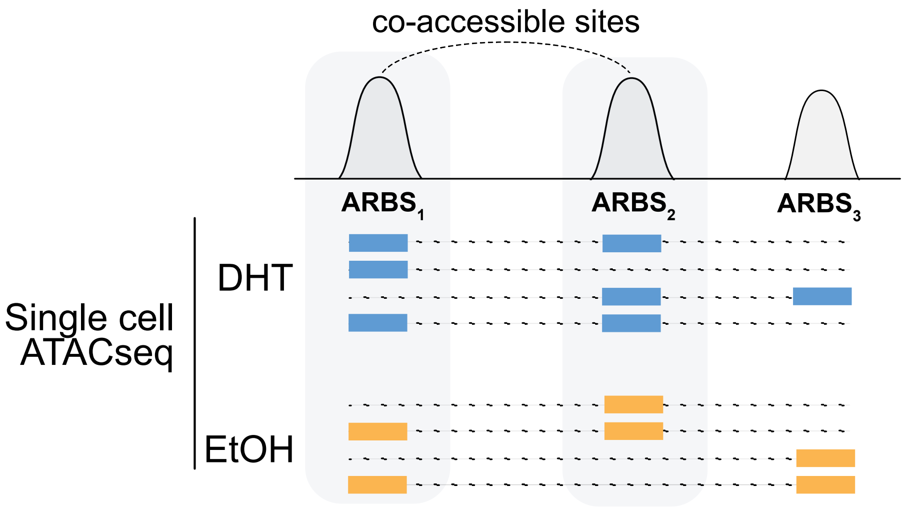

Figure S11: Schematic representation of co-accessibility quantification between ARBS using scATACseq data.

Figure S12

A

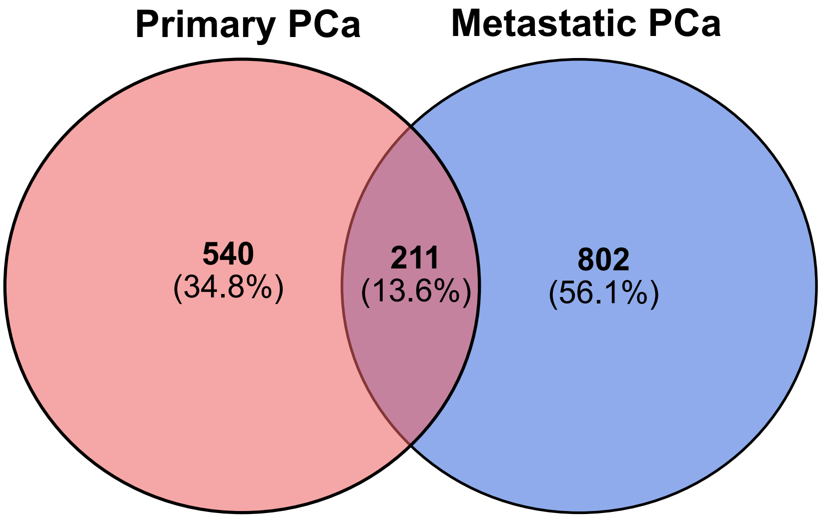

B

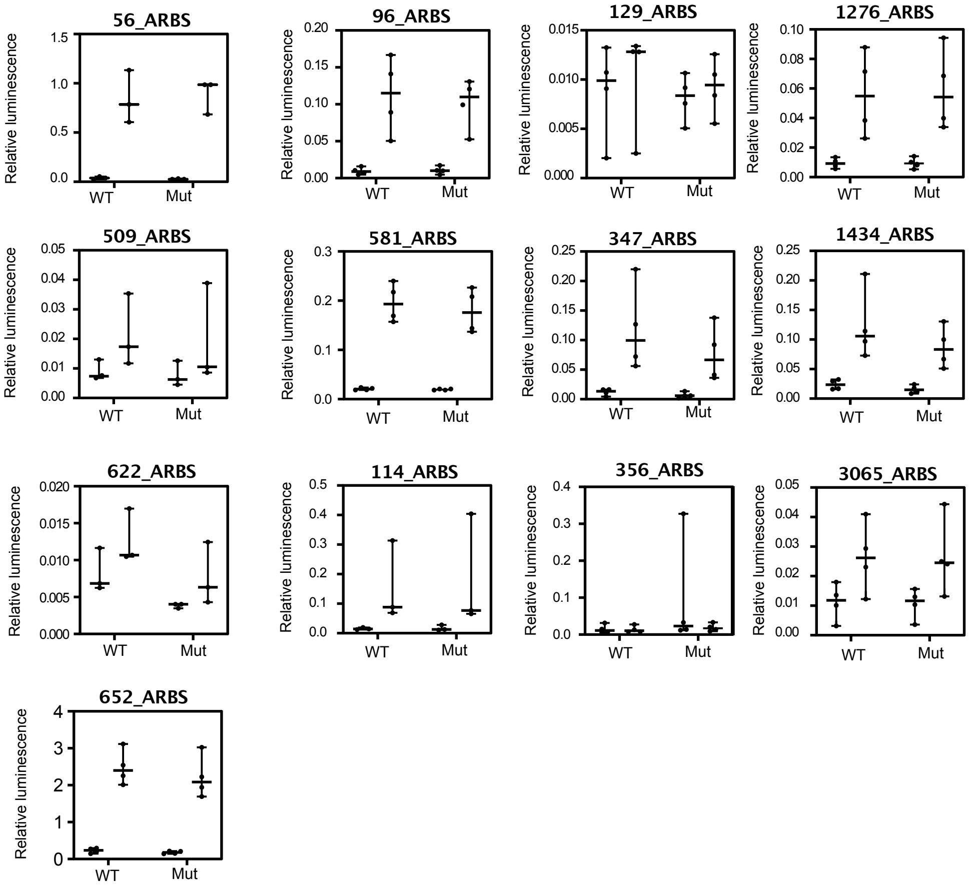

Figure S12: (A) Venn diagram of SNVs identified by WGS from primary PCa (n= 196) and metastatic CRPC (n=101). (B) Effects of SNV on the enhancer activity was quantified by the luciferase assay. From this 3/16 SNVs significantly affected androgen-mediated enhancer activity (Figure 6B). The remaining 13/16 SNVs did not significantly impact enhancer activity (p-value>0.05, 3 biological replica±SD).

Figure S13

A

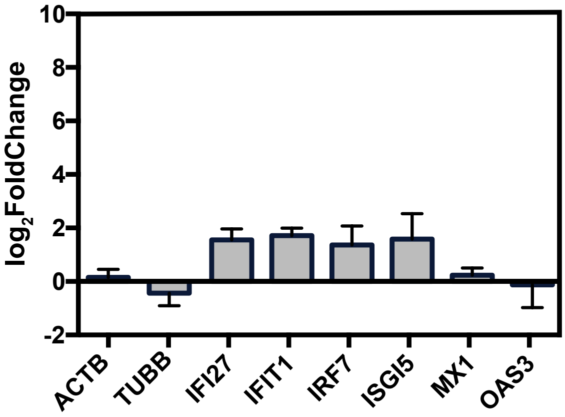

B

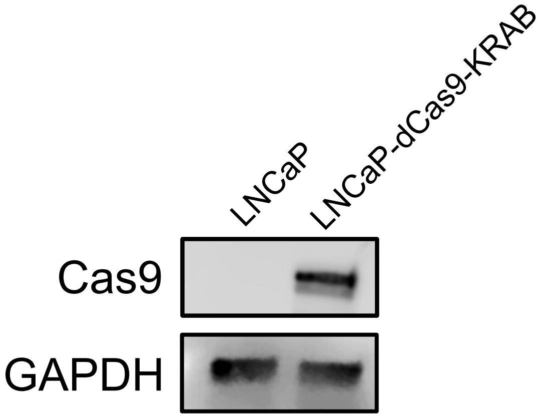

Figure S13: (A) The expression of genes involved in the IFNγ signaling pathways quantified by qRT-PCR in LNCaP cells transfected with the ARBS STARRseq library. The bar graph shows the mean expression with standard-deviation from 3 biological replicates. **(B)** Lysates form LNCaP cells with and without stably expressing dCas-KRAB were separated on SDS-PAGE and probed with antibody against Cas9. B-actin was used as the loading control.

Figure S14

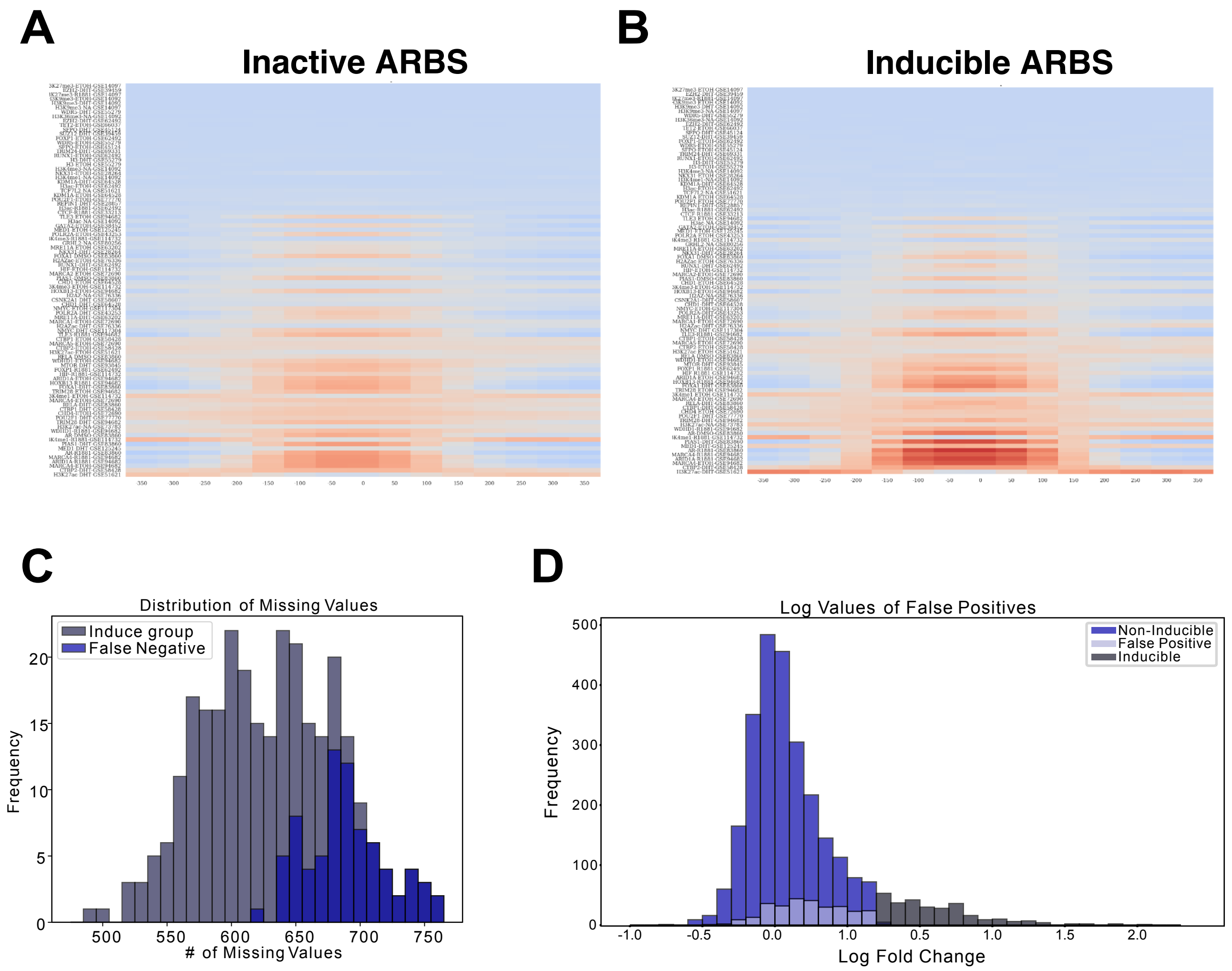

Figure S14: Heatmap of average occupancy score (50 bp resolution) for inducible and inactive AR enhancers in EtOH **(A)** and DHT **(B)** treated LNCaP cells. Factors with a below-average occupancy score are shown in blue while those with an above-average occupancy score are shown in red. **(C)** Distribution of missing values in ChIPseq data for all inducible regions and those misclassified inducible regions. The x-axis represents the number of missing values within a 50bp region. Misclassified inducible regions have a larger proportion of missing values, leading to poorly defined inputs to the classifier. **(D)** Distribution of androgen induced STARRseq expression for regions that were classified as inactive, inducible, and falsely predicted to be inducible by the classifier. The distribution is both skewed towards positive values and close to the cutoff for the inducible group leading to incorrect predictions.
