# Supplementary material for: Functional mapping of androgen receptor enhancer activity
Source: Genome Biol. 2021 May 11;22:149. doi: 10.1186/s13059-021-02339-6 (PMC8112059; doi:10.1186/s13059-021-02339-6)
Supplement: Supplementary file 2 — Additional file 2. All the publicly available data used. [file 13059_2021_2339_MOESM2_ESM.docx]

| Experiment | Feature | GEO | Reference |
| --- | --- | --- | --- |
| ATAC-seq | Chromatin compaction | GSE148925 | [[1]](https://paperpile.com/c/54kwbG/2sES) |
| ChIPseq | H3K4me1 | GSE114732 | [[2]](https://paperpile.com/c/54kwbG/oyKU) |
| ChIPseq | H3K4me3 | GSE114732 | [[2]](https://paperpile.com/c/54kwbG/oyKU) |
| ChIPseq | HIF | GSE114732 | [[2]](https://paperpile.com/c/54kwbG/oyKU) |
| ChIPseq | NMYC | GSE117304 | [[3]](https://paperpile.com/c/54kwbG/moxM) |
| ChIPseq | MED1 | GSE125245 | [[4]](https://paperpile.com/c/54kwbG/q290) |
| ChIPseq | H3ac | GSE14092 | [[5]](https://paperpile.com/c/54kwbG/aH38) |
| ChIPseq | H3K36me3 | GSE14092 | [[5]](https://paperpile.com/c/54kwbG/aH38) |
| ChIPseq | H3K4me1 | GSE14092 | [[5]](https://paperpile.com/c/54kwbG/aH38) |
| ChIPseq | H3K4me3 | GSE14092 | [[5]](https://paperpile.com/c/54kwbG/aH38) |
| ChIPseq | H3K9me3 | GSE14092 | [[5]](https://paperpile.com/c/54kwbG/aH38) |
| ChIPseq | H3K27me3 | GSE14097 | [[5]](https://paperpile.com/c/54kwbG/aH38) |
| ChIPseq | H3K9me3 | GSE14097 | [[5]](https://paperpile.com/c/54kwbG/aH38) |
| ChIPseq | POL2 | GSE28126 | [[6]](https://paperpile.com/c/54kwbG/hHw0) |
| ChIPseq | NKX31 | GSE28264 | [[7]](https://paperpile.com/c/54kwbG/TVvE) |
| ChIPseq | REPIN1 | GSE28857 | [[8]](https://paperpile.com/c/54kwbG/qtBQ) |
| ChIPseq | CTCF | GSE33213 |  |
| ChIPseq | GATA2 | GSE38452 |  |
| ChIPseq | EZH2 | GSE39459 | [[9]](https://paperpile.com/c/54kwbG/Iugb) |
| ChIPseq | SUZ12 | GSE39459 | [[9]](https://paperpile.com/c/54kwbG/Iugb) |
| ChIPseq | PSF | GSE45124 | [[10]](https://paperpile.com/c/54kwbG/3WCR) |
| ChIPseq | H3K27ac | GSE51621 | [[11]](https://paperpile.com/c/54kwbG/U2qo) |
| ChIPseq | TCF7L2 | GSE51621 | [[11]](https://paperpile.com/c/54kwbG/U2qo) |
| ChIAPET | AR | GSE54946 | [[12]](https://paperpile.com/c/54kwbG/cftx) |
| ChIPseq | H3 | GSE55279 | [[13]](https://paperpile.com/c/54kwbG/QnSb) |
| ChIPseq | WDR5 | GSE55279 | [[13]](https://paperpile.com/c/54kwbG/QnSb) |
| ChIPseq | CTBP1 | GSE58428 | [[14]](https://paperpile.com/c/54kwbG/owkD) |
| ChIPseq | CTBP2 | GSE58428 | [[14]](https://paperpile.com/c/54kwbG/owkD) |
| ChIPseq | CSNK2A1 | GSE58607 | [[15]](https://paperpile.com/c/54kwbG/yQmn) |
| ChIPseq | EZH2 | GSE62492 | [[16]](https://paperpile.com/c/54kwbG/Dkkz) |
| ChIPseq | FOXP1 | GSE62492 | [[16]](https://paperpile.com/c/54kwbG/Dkkz) |
| ChIPseq | H3ac | GSE62492 | [[16]](https://paperpile.com/c/54kwbG/Dkkz) |
| ChIPseq | RUNX1 | GSE62492 | [[16]](https://paperpile.com/c/54kwbG/Dkkz) |
| ChIPseq | MRE11A | GSE63202 | [[17]](https://paperpile.com/c/54kwbG/LBqw) |
| ChIPseq | CHD1 | GSE64528 | [[18]](https://paperpile.com/c/54kwbG/TJGk) |
| ChIPseq | KDM1A | GSE64528 | [[18]](https://paperpile.com/c/54kwbG/TJGk) |
| RNAseq | RNA | GSE64530 | [[18]](https://paperpile.com/c/54kwbG/TJGk) |
| ChIPseq | TET2 | GSE66037 | [[19]](https://paperpile.com/c/54kwbG/wdUe) |
| ChIPseq | TRIM24 | GSE69331 | [[20]](https://paperpile.com/c/54kwbG/lvDK) |
| ChIPseq | CHD4 | GSE72690 | [[21]](https://paperpile.com/c/54kwbG/66FO) |
| ChIPseq | SMARCA1 | GSE72690 | [[21]](https://paperpile.com/c/54kwbG/66FO) |
| ChIPseq | SMARCA2 | GSE72690 | [[21]](https://paperpile.com/c/54kwbG/66FO) |
| ChIPseq | SMARCA4 | GSE72690 | [[21]](https://paperpile.com/c/54kwbG/66FO) |
| ChIPseq | SMARCA5 | GSE72690 | [[21]](https://paperpile.com/c/54kwbG/66FO) |
| ChIPseq | H3K27ac | GSE73783 | [[22]](https://paperpile.com/c/54kwbG/Nman) |
| ChIPseq | H2AZac | GSE76336 | [[23]](https://paperpile.com/c/54kwbG/CzaB) |
| ChIPseq | H2AZ | GSE76336 | [[23]](https://paperpile.com/c/54kwbG/CzaB) |
| ChIPseq | POU2F1 | GSE77770 | [[24]](https://paperpile.com/c/54kwbG/sPYE) |
| ChIPseq | GRHL2 | GSE80256 | [[25]](https://paperpile.com/c/54kwbG/juWH) |
| ChIPseq | AR | GSE83860,GSE148358,GSE84432 | [[26–28]](https://paperpile.com/c/54kwbG/X8WX+tgNX+or5z) |
| GROseq | Nascent RNA | GSE83860/GSE84432 | [[26,28]](https://paperpile.com/c/54kwbG/X8WX+or5z) |
| ChIPseq | FOXA1 | GSE83860 | [[26]](https://paperpile.com/c/54kwbG/X8WX) |
| ChIPseq | PIAS1 | GSE83860 | [[26]](https://paperpile.com/c/54kwbG/X8WX) |
| ChIPseq | RELA | GSE83860 | [[26]](https://paperpile.com/c/54kwbG/X8WX) |
| ChIPseq | MTOR | GSE93845 | [[29]](https://paperpile.com/c/54kwbG/hbKe) |
| ChIPseq | HOXB13 | GSE94682 | [[30]](https://paperpile.com/c/54kwbG/OB53) |
| ChIPseq | ARID1A | GSE94682 | [[30]](https://paperpile.com/c/54kwbG/OB53) |
| ChIPseq | SMARCA4 | GSE94682 | [[30]](https://paperpile.com/c/54kwbG/OB53) |
| ChIPseq | TLE3 | GSE94682 | [[30]](https://paperpile.com/c/54kwbG/OB53) |
| ChIPseq | TRIM28 | GSE94682 | [[30]](https://paperpile.com/c/54kwbG/OB53) |
| ChIPseq | WDHD1 | GSE94682 | [[30]](https://paperpile.com/c/54kwbG/OB53) |

**Bibliography**

[1. Gao S, Chen S, Han D, Wang Z, Li M, Han W, et al. Chromatin binding of FOXA1 is promoted by LSD1-mediated demethylation in prostate cancer. Nat Genet. 2020;52:1011–7.](http://paperpile.com/b/54kwbG/2sES)

[2. Tran MGB, Bibby BAS, Yang L, Lo F, Warren AY, Shukla D, et al. Independence of HIF1a and androgen signaling pathways in prostate cancer. BMC Cancer. 2020;20:469.](http://paperpile.com/b/54kwbG/oyKU)

[3. Berger A, Brady NJ, Bareja R, Robinson B, Conteduca V, Augello MA, et al. N-Myc-mediated epigenetic reprogramming drives lineage plasticity in advanced prostate cancer. J Clin Invest. 2019;129:3924–40.](http://paperpile.com/b/54kwbG/moxM)

[4. Rasool RU, Natesan R, Deng Q, Aras S, Lal P, Sander Effron S, et al. CDK7 Inhibition Suppresses Castration-Resistant Prostate Cancer through MED1 Inactivation. Cancer Discov. 2019;9:1538–55.](http://paperpile.com/b/54kwbG/q290)

[5. Yu J, Yu J, Mani R-S, Cao Q, Brenner CJ, Cao X, et al. An integrated network of androgen receptor, polycomb, and TMPRSS2-ERG gene fusions in prostate cancer progression. Cancer Cell. 2010;17:443–54.](http://paperpile.com/b/54kwbG/aH38)

[6. Massie CE, Lynch A, Ramos-Montoya A, Boren J, Stark R, Fazli L, et al. The androgen receptor fuels prostate cancer by regulating central metabolism and biosynthesis. EMBO J. 2011;30:2719–33.](http://paperpile.com/b/54kwbG/hHw0)

[7. Tan PY, Chang CW, Chng KR, Wansa KDSA, Sung W-K, Cheung E. Integration of regulatory networks by NKX3-1 promotes androgen-dependent prostate cancer survival. Mol Cell Biol. 2012;32:399–414.](http://paperpile.com/b/54kwbG/TVvE)

[8. Zhang Z, Chang CW, Goh WL, Sung W-K, Cheung E. CENTDIST: discovery of co-associated factors by motif distribution. Nucleic Acids Res. 2011;39:W391–9.](http://paperpile.com/b/54kwbG/qtBQ)

[9. Xu K, Wu ZJ, Groner AC, He HH, Cai C, Lis RT, et al. EZH2 oncogenic activity in castration-resistant prostate cancer cells is Polycomb-independent. Science. 2012;338:1465–9.](http://paperpile.com/b/54kwbG/Iugb)

[10. Takayama K-I, Horie-Inoue K, Katayama S, Suzuki T, Tsutsumi S, Ikeda K, et al. Androgen-responsive long noncoding RNA CTBP1-AS promotes prostate cancer. EMBO J. 2013;32:1665–80.](http://paperpile.com/b/54kwbG/3WCR)

[11. Hazelett DJ, Rhie SK, Gaddis M, Yan C, Lakeland DL, Coetzee SG, et al. Comprehensive functional annotation of 77 prostate cancer risk loci. PLoS Genet. 2014;10:e1004102.](http://paperpile.com/b/54kwbG/U2qo)

[12. Zhang Z, Chng KR, Lingadahalli S, Chen Z, Liu MH, Do HH, et al. An AR-ERG transcriptional signature defined by long-range chromatin interactomes in prostate cancer cells. Genome Res. 2019;29:223–35.](http://paperpile.com/b/54kwbG/cftx)

[13. Kim J-Y, Banerjee T, Vinckevicius A, Luo Q, Parker JB, Baker MR, et al. A role for WDR5 in integrating threonine 11 phosphorylation to lysine 4 methylation on histone H3 during androgen signaling and in prostate cancer. Mol Cell. 2014;54:613–25.](http://paperpile.com/b/54kwbG/QnSb)

[14. Takayama K-I, Suzuki T, Fujimura T, Urano T, Takahashi S, Homma Y, et al. CtBP2 modulates the androgen receptor to promote prostate cancer progression. Cancer Res. 2014;74:6542–53.](http://paperpile.com/b/54kwbG/owkD)

[15. Basnet H, Su XB, Tan Y, Meisenhelder J, Merkurjev D, Ohgi KA, et al. Tyrosine phosphorylation of histone H2A by CK2 regulates transcriptional elongation. Nature. 2014;516:267–71.](http://paperpile.com/b/54kwbG/yQmn)

[16. Takayama K-I, Suzuki T, Tsutsumi S, Fujimura T, Urano T, Takahashi S, et al. RUNX1, an androgen- and EZH2-regulated gene, has differential roles in AR-dependent and -independent prostate cancer. Oncotarget. 2015;6:2263–76.](http://paperpile.com/b/54kwbG/Dkkz)

[17. Puc J, Kozbial P, Li W, Tan Y, Liu Z, Suter T, et al. Ligand-dependent enhancer activation regulated by topoisomerase-I activity. Cell. 2015;160:367–80.](http://paperpile.com/b/54kwbG/LBqw)

[18. Metzger E, Willmann D, McMillan J, Forne I, Metzger P, Gerhardt S, et al. Assembly of methylated KDM1A and CHD1 drives androgen receptor-dependent transcription and translocation. Nat Struct Mol Biol. 2016;23:132–9.](http://paperpile.com/b/54kwbG/TJGk)

[19. Takayama K-I, Misawa A, Suzuki T, Takagi K, Hayashizaki Y, Fujimura T, et al. TET2 repression by androgen hormone regulates global hydroxymethylation status and prostate cancer progression. Nat Commun. 2015;6:8219.](http://paperpile.com/b/54kwbG/wdUe)

[20. Groner AC, Cato L, de Tribolet-Hardy J, Bernasocchi T, Janouskova H, Melchers D, et al. TRIM24 Is an Oncogenic Transcriptional Activator in Prostate Cancer. Cancer Cell. 2016;29:846–58.](http://paperpile.com/b/54kwbG/lvDK)

[21. Ye Z, Chen Z, Sunkel B, Frietze S, Huang TH-M, Wang Q, et al. Genome-wide analysis reveals positional-nucleosome-oriented binding pattern of pioneer factor FOXA1. Nucleic Acids Res. 2016;44:7540–54.](http://paperpile.com/b/54kwbG/66FO)

[22. Taberlay PC, Achinger-Kawecka J, Lun ATL, Buske FA, Sabir K, Gould CM, et al. Three-dimensional disorganization of the cancer genome occurs coincident with long-range genetic and epigenetic alterations. Genome Res. 2016;26:719–31.](http://paperpile.com/b/54kwbG/Nman)

[23. Valdés-Mora F, Gould CM, Colino-Sanguino Y, Qu W, Song JZ, Taylor KM, et al. Acetylated histone variant H2A.Z is involved in the activation of neo-enhancers in prostate cancer. Nat Commun. 2017;8:1346.](http://paperpile.com/b/54kwbG/CzaB)

[24. Obinata D, Takayama K, Fujiwara K, Suzuki T, Tsutsumi S, Fukuda N, et al. Targeting Oct1 genomic function inhibits androgen receptor signaling and castration-resistant prostate cancer growth. Oncogene. 2016;35:6350–8.](http://paperpile.com/b/54kwbG/sPYE)

[25. Paltoglou S, Das R, Townley SL, Hickey TE, Tarulli GA, Coutinho I, et al. Novel Androgen Receptor Coregulator GRHL2 Exerts Both Oncogenic and Antimetastatic Functions in Prostate Cancer. Cancer Res. 2017;77:3417–30.](http://paperpile.com/b/54kwbG/juWH)

[26. Malinen M, Niskanen EA, Kaikkonen MU, Palvimo JJ. Crosstalk between androgen and pro-inflammatory signaling remodels androgen receptor and NF-κB cistrome to reprogram the prostate cancer cell transcriptome. Nucleic Acids Res. 2017;45:619–30.](http://paperpile.com/b/54kwbG/X8WX)

[27. Baumgart SJ, Nevedomskaya E, Lesche R, Newman R, Mumberg D, Haendler B. Darolutamide antagonizes androgen signaling by blocking enhancer and super-enhancer activation. Mol Oncol. 2020;14:2022–39.](http://paperpile.com/b/54kwbG/tgNX)

[28. Toropainen S, Niskanen EA, Malinen M, Sutinen P, Kaikkonen MU, Palvimo JJ. Global analysis of transcription in castration-resistant prostate cancer cells uncovers active enhancers and direct androgen receptor targets. Sci Rep. 2016;6:33510.](http://paperpile.com/b/54kwbG/or5z)

[29. Audet-Walsh É, Dufour CR, Yee T, Zouanat FZ, Yan M, Kalloghlian G, et al. Nuclear mTOR acts as a transcriptional integrator of the androgen signaling pathway in prostate cancer. Genes Dev. 2017;31:1228–42.](http://paperpile.com/b/54kwbG/hbKe)

[30. Stelloo S, Nevedomskaya E, Kim Y, Hoekman L, Bleijerveld OB, Mirza T, et al. Endogenous androgen receptor proteomic profiling reveals genomic subcomplex involved in prostate tumorigenesis. Oncogene. 2018;37:313–22.](http://paperpile.com/b/54kwbG/OB53)
