# Supplementary material for: Functional mapping of androgen receptor enhancer activity
Source: Genome Biol. 2021 May 11;22:149. doi: 10.1186/s13059-021-02339-6 (PMC8112059; doi:10.1186/s13059-021-02339-6)
Supplement: Supplementary file 4 — Additional file 4. Review history. [file 13059_2021_2339_MOESM4_ESM.docx]

Review History

**First round of review**

**Reviewer 1**

**Are you able to assess all statistics in the manuscript, including the appropriateness of statistical tests used?**

No, I do not feel adequately qualified to assess the statistics.

**Comments to author:**

In the manuscript entitled "Functional mapping of androgen receptor enhancer activity in LNCaP prostate cancer cells" Huan and coworkers have generated by combining their Self-Transcribing Active Regulatory Regions sequencing (STARRseq) data with others' published data a functional map of AR enhancer activity. The data suggest that AR-driven inducible enhancers act as a regulatory hub that frequently cooperate with other AR binding sites (ARBS) to drive transcription. Interestingly, several clinical mutations to these enhancers frequently alter their activity.

Major issues:

The authors ignore or disregard in their results the influence of chromatin accessibility, or the role of AR in opening closed chromatin sites. Based on the prediction in Fig. 3A, chromatin remodeling complexes in androgen-exposed conditions predict well inducibility of ARBS. Also, AR-ARID1A-SMARCA4 in androgen-exposed conditions are good predictors (Supp. Fig. 9B) for inducibility. The authors utilize published DNase-seq data, but the data are not a good match for their system. The published DNaseq-seq data are from cells exposed to R1881 for 12 h compared to 4-h DHT exposure used by the authors. However, the authors have performed their scATAC-seq experiments with the same hormone conditions (DHT for 4 h). Regardless of the scATAC-seq data, the discussion of AR-inducible chromatin accessibility and enhancer activity is lacking. The authors should expand the influence of analysis/discussion of chromatin accessibility to the enhancer activity. This is important, as the chromatin remodeling complexes with AR are good predictors of inducibility. Finally, the authors could utilize TCGA ATAC-seq data from PRAD patients in their analysis.

The authors draw their conclusion from a single time point analysis (DHT for 4h). Thus, temporal kinetics of transcription factor-binding and target gene expression are ignored. It has been shown by GRO-seq that androgen regulated production of eRNA changes over time (see PMID: 27641228). Thus, many of the inactive enhancers could be misclassified due to the lack of temporal kinetic information. This could also be the reason why inactive enhancers have effects with CRISPRi in Fig. 4. The authors should either assess the temporal kinetics of enhancer activity, or clearly state the limitations of their analyses.

The utilization of public data is rather opaque. The authors give in most cases no credit (reference) to any of the studies that have generated the public datasets. They are mainly barely reported as accession numbers in the supplementary table. Furthermore, the authors remember to reference their own datasets in the text (e.g. ref 8) but not any other datasets (e.g. data in Figure 1). Credit where credit is due needs to be indicated.

Another opaque usage of public dataset is the AR ChIA-PET data. The authors fail to mention anywhere in the text that the ChIA-PET data are from VCaP prostate cancer cells, a very different cell line compared to LNCaP cells. VCaP cells has an amplification of wild-type receptor gene, leading to AR overexpression, while LNCaP cells express an LBD-point-mutated receptor. For this reviewer, it looks like the authors are trying to hide is information from the reader. This absolute needs to be rectified. The authors need to clearly indicate in the text and figure legends that the ChIA-PET data are from VCaP cells. Furthermore, the authors need to indicate the limitations of their results in this regard since the data is from entirely different cell line.

Minor issues:

The first reference to supplementary figure is supplementary fig.3. Figure should be reordered so that supplementary fig.1 is the first to be referenced.

The concept of shadow peaks discussed in the text is not a clear concept. The data presented by Glont et al. have been refuted by the Hager laboratory (see PMID: 31553919). This should either be removed, or the discussion expanded.

Some of the reference lack journal information, ref19 and ref45.

**Reviewer 2**

**Are you able to assess all statistics in the manuscript, including the appropriateness of statistical tests used?**

No, I do not feel adequately qualified to assess the statistics.

**Comments to author:**

The authors are attempting to address a common question in the field, namely why are there so many more active enhancers/TF binding sites for a particular TF than there are genes regulated by that TF. They use the method of STARR-seq (which uses a transfected reporter construct to measure effects on promoter activity due to cloned insert that has putative enhancer activity) to divide a set of AR binding sites into 3 classes (inducible activity, constitutive activity, and inactive. They find that the vast majority of the starting set of AR binding sites does not have any enhancer activity. There is no clear distinction between the functional vs nonfunctional sites.

Major issues

1)	They really cannot find any convincing characteristics that set the "functional" minority apart from the "nonfunctional" majority of sites except for AR ChIP-seq peak height. This raises the question as to whether their starting set of AR peaks was of high quality.

2)	The manuscript is very confusingly written, the supplementary figures are not in the order discussed in the text, and many experimental details have been left out. There are many cases throughout the manuscript where more description is needed as to what was done, it is not clear what is shown in a figure, there is missing info in the legend, etc.

3)	Figure 6 does not really fit with the theme of the rest of the paper.

4)	It is difficult to appreciate a body of work if the reader has to keep searching through the supplementary figures to understand the research. Please consider whether it would make a stronger paper to include some of the supplementary figures in the main text (in the order in which they are discussed!).

Other issues:

1)	The percentage of enhancers that are subsequently determined to be "functional" is heavily dependent on the starting collection of enhancers and I don't really understand what that set is composed of. It is not clear what is meant by "commonly observed clinical ARBS"? The text states that the sites were taken from "all normal tissue or primary PCa clinical AR ChIPseq". However, only one reference is given. I would think that many more AR ChIP-seq datasets have been produced. Also, it is not clear by the way the sentence is written whether these sites are selected because they are occupied by AR in both normal AND PCa AR ChIP-seq datasets or in normal OR PCa AR ChIP-seq datasets. More information about these starting datasets is needed.

2)	Figure 1A is not very useful. It is so tiny that it can't be interpreted. Also, the figure legend for 1A is just a title. The legend should explain what the protocol is. STARRseq is not commonly done in all labs and you need to explain to the readers how you get a peak on a browser from the transfected construct.

3)	Fig 1B. I don't understand how the figure legend fits with the figure. You have AR ChIP-seq labeled as the red track but the legend says it is the blue track.

4)	Fig 1C. Not enough information is provided. What are the 42 sequences that were used? Were their activities the most robust, intermediate, a range when compared to the entire set?

5)	Fig 1E- not necessary

6)	Fig 1F- you need to explain the color legend for GROseq.

7)	I don't understand this statement: "Surprisingly, none of the ARBS that were found only in clinical tissue but not LNCaP cells were inducible enhancers (n=867; Supplementary Figure 6A+B). This rate of inactivity is significantly less than expected compared to the clinical ARBS that overlap with LNCaP (p<2.1x10-16). As these regions lacked AR binding they were separated in subsequent analysis (no AR)." How can something that is defined as an ARBS (Androgen Receptor Binding Site) lack AR binding. Or does the sentence need to be clarified in the text?

8)	Can you explain the exact mechanism of action of ENZA? Does it prevent AR translocation to the nucleus? Does it allow binding of AR but block transactivation activity?

9)	Fig 2B. What is P1, P2, P3, P4? These should be described in the legend.

10)	The supplementary figures are not presented in the right order in the text; please renumber them so that they are presented in a logical order. Also, it would be very helpful if the legends were on the same page as the figures.

11)	The authors state that "During optimization we found that ARBS inserts less than 250bp had marginal activity suggesting that the flanking sequences contribute to AR enhancer activity (Supplementary Figure 3).". However, it appears as if only one site was actually tested in Sup Fig 3. This is not sufficient evidence to make a strong conclusion as to the requirement for flanking sequences. Also, where is the motif in the fragment?

12)	Sup Fig 4b. I am not sure I understand the significance of these plots. What point are you making?

13)	Figure 3. You state that the peak height of AR is the best predictor. Does this mean that the AR ChIP-seq peak set you started with may have not been of great quality, containing many small (false positive?) peaks? Perhaps a high false positive ChIP-seq dataset explains your statement "From this we found that only a fraction of ARBS (7%) showed androgen-dependent enhancer activity, while the majority (81%) were inactive"?

14)	Basically, I don't think that figure 4 provides much insight into how to identify a critical enhancer. The results obtained after inactivation of the different enhancer types are not consistent.

15)	Fig 6D. You do not define NT and you don't really explain what the experiment was that you have shown in the figure.

16)	For the CRISPRi experiments, did you monitor the genomic region to see if the expected epigenetic modifications occurred?

17)	You make this statement in the Discussion: "By systematically testing all clinical ARBS for enhancer activity, this provides the first detailed "map" needed to investigate these clinically important problems.

a) I note that you have only done these experiments in one prostate cancer cell line. Because of the very low rate of identification of "functional" enhancers in your experiment, it could easily be that you would get a completely different set if you used a different prostate cancer cell line. Also, what if you used a second promoter, would you have identified a different set of functional enhancers? If so, you do not yet have a detailed map.

b) As stated earlier, the composition of your starting set of ARBS will affect the percentages in your functional experiments. You need to be sure that you have a high quality, comprehensive set of AR ChIP-seq data. It is really difficult to know if this is the case from your description of the datasets. The fact that the height of the AR peak is the best predictor may indicate that you have poor quality peaks in your set of peaks.

**Authors Response**

**Point-by-point responses to the reviewers’ comments:**

Reviewer #1:

Reviewer comment: The authors ignore or disregard in their results the influence of chromatin accessibility, or the role of AR in opening closed chromatin sites. Based on the prediction in Fig. 3A, chromatin remodeling complexes in androgen-exposed conditions predict well inducibility of ARBS. Also, AR-ARID1A-SMARCA4 in androgen-exposed conditions are good predictors (Supp. Fig. 9B) for inducibility. The authors utilize published DNase-seq data, but the data are not a good match for their system. The published DNaseq-seq data are from cells exposed to R1881 for 12 h compared to 4-h DHT exposure used by the authors. However, the authors have performed their scATAC-seq experiments with the same hormone conditions (DHT for 4 h). Regardless of the scATAC-seq data, the discussion of AR-inducible chromatin accessibility and enhancer activity is lacking. The authors should expand the influence of analysis/discussion of chromatin accessibility to the enhancer activity. This is important, as the chromatin remodeling complexes with AR are good predictors of inducibility. Finally, the authors could utilize TCGA ATAC-seq data from PRAD patients in their analysis.

*Author response: We would like to thank the reviewer for bringing up this important point. With our machine learning model, members of the SWI/SNF complex including ARID1A and SMARCA4 were predictive of inducible enhancers. This is not likely due to alterations in chromatin accessibility as STARRseq tests the enhancer capacity of a genomic region using ectopic plasmid that have marginal nucleosome binding and are not impacted by endogenous chromatin modifications. Demonstrating this, we observed very strong STARRseq signal in our non-AR driven positive controls regardless if they were hetero- or euchromatin in LNCaP cells (Supplementary Figure 3). Further, while both ARID1A and SMARCA4 binding are good predictors of inducible enhancers this was not simply due to increased accessibility. In our machine learning models we included both LNCaP ATACseq and DHS but did not see any increase in precision or accuracy. Potentially this may be due to the type of sites that we are characterizing with STARRseq. By focusing on common AR binding sites that are by definition accessible in prostate cancer we have largely removed accessibility as a variable. In support of this, when we compared TCGA ATAC-seq data from several PRAD patients almost all ARBS tested were found at euchromatin (>93% overall). Chromatin accessibility is a prerequisite for transcription factor binding (PMID: 29272704, 25552417, 26259101, 22508765, and 30996347) but this alone does not dictate enhancer activity. We have better clarified the text to both describe our work with ATACseq and DHS (page 5) and also the impact of accessibility in our discussion (page 12).*

*As suggested, we also characterized a published LNCaP ATACseq data with 4h DHT treatment (GSE148925) and found no difference in the results from that of 12h DHT treatment (Supplementary Figure 3).*

Reviewer comment: The authors draw their conclusion from a single time point analysis (DHT for 4h). Thus, temporal kinetics of transcription factor-binding and target gene expression are ignored. It has been shown by GRO-seq that androgen regulated production of eRNA changes over time (see PMID: 27641228). Thus, many of the inactive enhancers could be misclassified due to the lack of temporal kinetic information. This could also be the reason why inactive enhancers have effects with CRISPRi in Fig. 4. The authors should either assess the temporal kinetics of enhancer activity, or clearly state the limitations of their analyses.

*Author response: This is an important point and something the authors extensively discussed as relative activity could be influenced by temporal kinetics. In the end we chose to limit our study to a single early time point to allow us to focus solely on AR-mediated activity. Testing later time points may show altered enhancer activity, but it would be extremely difficult to separate direct AR enhancer activity from the potential impact of those genes that are upregulated/downregulated by androgen treatment. However, despite testing only a single time point, our results strongly correlate with steady-state clinical AR enhancer activity. In matched tumour samples from neoadjuvant enzalutamide trial H3K27Ac at inducible enhancers was significantly reduced when the AR was inhibited (post-enzalutamide treatment). This was not seen in either inactive or constitutive enhancers suggesting that even a single time point is strongly reflective of the AR enhancer activity. Finally, when we looked at GR STARRseq that had been tested in multiple time points (PMID: 30575722) there was a marginal change in the number of inducible enhancers identified at 4, 8 and 12hr following hormone treatment (4.9, 8.0 and 8.6% of GR binding sites, respectively). We have better described the limitation of our study on page 11.*

Reviewer comment: The utilization of public data is rather opaque. The authors give in most cases no credit (reference) to any of the studies that have generated the public datasets. They are mainly barely reported as accession numbers in the supplementary table. Furthermore, the authors remember to reference their own datasets in the text (e.g. ref 8) but not any other datasets (e.g. data in Figure 1). Credit where credit is due needs to be indicated.

*Author response: We would like to thank the reviewer for this comment. In no way did we want to diminish the importance of the work we utilized. The availability of public dataset dramatically improved the quality of our research and allowed us to investigate questions that would not have been possible due to technical or financial limitations. Access to this data was absolutely essential and highlights the value of sharing datasets. We should have done a better job acknowledging previous work in the initial draft. To better highlight the importance of the data used in this study, we have included citations and GEO accession numbers for all datasets in both the supplementary table and main text.*

Reviewer comment: Another opaque usage of public dataset is the AR ChIA-PET data. The authors fail to mention anywhere in the text that the ChIA-PET data are from VCaP prostate cancer cells, a very different cell line compared to LNCaP cells. VCaP cells has an amplification of wild-type receptor gene, leading to AR overexpression, while LNCaP cells express an LBD-point-mutated receptor. For this reviewer, it looks like the authors are trying to hide is information from the reader. This absolute needs to be rectified. The authors need to clearly indicate in the text and figure legends that the ChIA-PET data are from VCaP cells. Furthermore, the authors need to indicate the limitations of their results in this regard since the data is from entirely different cell line.

*Author response: This is an important point the reviewer highlighted. Unfortunately there is currently no publicly available AR ChIA-PET or HiChIP in LNCaP cells and HiC, which is available but with a 40kb window, does not have sufficient resolution to characterize enhancer-promoter connections among closely located ARBS. However, before working with the VCaP ChIA-PET data we compared the clinical ARBS tested in LNCaP cells with chromatin loops in VCaP cells and saw significant overlap (2451/3251). Further, when looking at other enhancer associated histone marks like H3K27Ac in VCaP we observed an overall similar trend to that observed with LNCaP, giving us greater confidence in the concordance of these two models (Supplementary Figure 8). Due to this limitation of this model we also characterized the co-accessibility in LNCaP from scATACseq (Figure 5D) which can be used as surrogate for chromatin looping (PMID: 30078726). To better highlight the shortcomings we have described the source and limitations of our data in the text (page 8).*

Reviewer comment: The first reference to supplementary figure is supplementary fig.3. Figure should be reordered so that supplementary fig.1 is the first to be referenced.

*Author response: This is an excellent suggestion that was made by both reviewers. We have reordered our supplementary figures so that Supplementary Figure 1 is referenced first in the result section.*

Reviewer comment: The concept of shadow peaks discussed in the text is not a clear concept. The data presented by Glont et al. have been refuted by the Hager laboratory (see PMID: 315573919). This should either be removed, or the discussion expanded.

*Author response: Thank you for highlighting this point. Our results correlate with that of the Hager laboratory as we found that these genomic regions are evolutionary conserved. To avoid this controversial hypothesis we have removed this sentence from the manuscript.*

Reviewer comment: Some of the reference lack journal information, ref19 and ref45.

*Author response: We thank the reviewer for pointing out this error. The references have been corrected.*

Reviewer #2:

Reviewer comment: They really cannot find any convincing characteristics that set the "functional" minority apart from the "nonfunctional" majority of sites except for AR ChIP-seq peak height. This raises the question as to whether their starting set of AR peaks was of high quality.

*Author response: Thank you for bringing up this important point. Given the potential heterogeneity of individual AR cistromes we also were very concerned about this issue when starting this study. To avoid testing rare or uncommon AR bindings (ARBS) we used a very conservative selection criteria. Utilizing data from a large clinical ChIPseq (PMID: 26457646) study, we chose to characterize only those sites that were found in all normal prostate samples (n=7), PCa tumours (n=13) or both. Supporting our annotation we observed excellent cross-validation of both H3K27Ac and AR in a recently published large (n=100) ChIPseq dataset (PMID: 30464211). Further, while AR ChIPseq peak height was the strongest predictor of inducible enhancer activity we also observed that additional histone marks and transcription factors including PIAS1, ARID1A, H3K27Ac and MED1 had very strong binding energy in our model. Supporting that enhancer activity wasn’t solely due to AR peak height, we identified many inactive ARBS (196/2479) that had greater AR peak height than that of the median “functional”/inducible enhancer. Finally, we observed equivalent evolutionary conservation across all ARBS classes (Figure 4 H) suggesting that these inactive sites represent conserved CRE. Overall we are confident that our starting set of AR binding sites was of very high quality. To clarify our selection process we have better described in the text how we selected these binding sites on page 4. We have also expanded the discussion on why AR peak height is not the only defining factor of enhancer activity in page 12.*

Reviewer comment: The manuscript is very confusingly written, the supplementary figures are not in the order discussed in the text, and many experimental details have been left out. There are many cases throughout the manuscript where more description is needed as to what was done, it is not clear what is shown in a figure, there is missing info in the legend, etc.

*Author response: Thank you for the suggestion. To improve the readability of this work we have increased the experimental details in both figure legends and text to better describe what was done. These changes can be seen throughout the manuscript. Further, we have included the figure legend in all supplementary figures.*

Reviewer comment: Figure 6 does not really fit with the theme of the rest of the paper.

*Author response: While less mechanism-driven than the remainder of this work, we feel that Figure 6 does demonstrate the potential translational utility of this work. There is increasing evidence that non-coding mutations can act as drivers of growth and resistance in prostate cancer (PMID: 31974375 and 29909987). However most of these somatic mutations have not been tested due to poor functional characterization of cis-regulatory elements. By focusing only on those mutations at inducible AR enhancers that act as regulatory “hubs” we can efficiently identify potentially important mutations. We have changed the text to better describe the utility of the method (page 10)*

Reviewer comment: It is difficult to appreciate a body of work if the reader has to keep searching through the supplementary figures to understand the research. Please consider whether it would make a stronger paper to include some of the supplementary figures in the main text (in the order in which they are discussed!).

*Author response: We would like to thank the reviewer for this suggestion. We have renumbered the supplementary figures to make it easier to read and incorporated some of this data into the main text.*

Reviewer comment: The percentage of enhancers that are subsequently determined to be "functional" is heavily dependent on the starting collection of enhancers and I don't really understand what that set is composed of. It is not clear what is meant by "commonly observed clinical ARBS"? The text states that the sites were taken from "all normal tissue or primary PCa clinical AR ChIPseq". However, only one reference is given. I would think that many more AR ChIP-seq datasets have been produced. Also, it is not clear by the way the sentence is written whether these sites are selected because they are occupied by AR in both normal AND PCa AR ChIP-seq datasets or in normal OR PCa AR ChIP-seq datasets. More information about these starting datasets is needed.

*Author response: Thank you for this very important point. We have clarified the text to better describe our selection criteria and the starting datasets (page 4).*

Reviewer comment: Figure 1A is not very useful. It is so tiny that it can't be interpreted. Also, the figure legend for 1A is just a title. The legend should explain what the protocol is. STARRseq is not commonly done in all labs and you need to explain to the readers how you get a peak on a browser from the transfected construct.

*Author response: This is an excellent suggestion. To better explain the protocol we have increased the size of Figure 1A but more importantly incorporated a better description of the protocol in both the figure legend and the main text (pages 4 and 30).*

Reviewer comment: Fig 1B. I don't understand how the figure legend fits with the figure. You have AR ChIP-seq labeled as the red track but the legend says it is the blue track.

*Author response: We would like to thank the reviewer for finding the error in our figure legends. We have now corrected this and correctly labeled the ChIPseq and STARRseq tracks (page 30).*

Reviewer comment: Fig 1C. Not enough information is provided. What are the 42 sequences that were used? Were their activities the most robust, intermediate, a range when compared to the entire set?

*Author response: Those regions that were functionally validated with a luciferase assay were randomly selected and represent lowly, intermediate and highly active inducible enhancers. We chose to validate different levels of activity to compare these two orthogonal approaches. The rate of correlation we observed between STARRseq and luciferase reporters is very similar to published work (PMID: 27565349 and 23328393). We have better described our selection criteria for validation on page 4.*

Reviewer comment: Fig 1E- not necessary

*Author response: We chose to introduce this graphical representation to help the reader understand the functional differences between the different enhancer classes. For readers who may be less familiar with either enhancers or the STARRseq method, we feel that this can help clarify the definitions used in this study.*

Reviewer comment: Fig 1F- you need to explain the color legend for GROseq.

*Author response: We have included in the figure legends a more detailed explanation of the colour legend for the public GROseq and ChIPseq data (page 30).*

Reviewer comment: I don't understand this statement:"Surprisingly, none of the ARBS that were found only in clinical tissue but not LNCaP cells were inducible enhancers (n=867; Supplementary Figure 6A+B). This rate of inactivity is significantly less than expected compared to the clinical ARBS that overlap with LNCaP (p<2.1x10-16). As these regions lacked AR binding they were separated in subsequent analysis (no AR)." How can something that is defined as an ARBS (Androgen Receptor Binding Site) lack AR binding. Or does the sentence need to be clarified in the text?

*Author response: This refers to those common ARBS that were present in the AR cistrome of clinical tissue but not LNCaP cells. We have clarified the text to better explain this difference (page 5).*

Reviewer comment: Can you explain the exact mechanism of action of ENZA? Does it prevent AR translocation to the nucleus? Does it allow binding of AR but block transactivation activity?

*Author response: Thank you for the suggestion. ENZA is an antiandrogen that binds directly to the AR ligand binding site and prevents both androgen-mediated activation and nuclear translocation (PMID: 19359544). Our clinical results with ENZA are very important to this study as they allow us to characterize enhancer histone marks in the presence (pre-treatment) or absence (post-treatment) of transcriptionally active AR. Confirming our in vitro STARRseq annotation, we observed no change in AR-mediated H3K27Ac levels at constitutive or inactive ARBS while there was a significant decrease at inducible sites. This gives much greater confidence in our STARRseq results as they correlate with steady state AR activity. We have included a description of the ENZA mechanism of action and better described the importance of these clinical results on page 6.*

Reviewer comment: Fig 2B. What is P1, P2, P3, P4? These should be described in the legend.

*Author response: P1-P4 refers to the individual patient tumours that we conduced H3K27Ac ChIPseq. We have corrected both the figure and legend to better describe this (page 30)*

Reviewer comment: The supplementary figures are not presented in the right order in the text; please renumber them so that they are presented in a logical order. Also, it would be very helpful if the legends were on the same page as the figures.

*Author response: We thank both the reviewers for highlighting this. We have now rectified this and the legends for the supplementary figures are now included on the same page.*

Reviewer comment: The authors state that "During optimization we found that ARBS inserts less than 250bp had marginal activity suggesting that the flanking sequences contribute to AR enhancer activity (Supplementary Figure 3).". However, it appears as if only one site was actually tested in Sup Fig 3. This is not sufficient evidence to make a strong conclusion as to the requirement for flanking sequences. Also, where is the motif in the fragment?

*Author response: The importance of larger fragments for enhancer assays is supported through several pieces of evidence including our initial optimization and other published STARRseq papers where they observed an increase in glucocorticoid receptor activity with larger inserts (PMID: 27565349). Further in a recent study that tested the impact of insert size on enhancer activity in massively multi-parallel reporter assays (n=2,440), the authors demonstrated that larger sequences (>500bp) can be critical for functional activity (PMID: 33046894).*

*ARE motif is central to the AREIII enhancer region tested. We have included the motif location in the figure to better demonstrate this. (Supplementary Figure 1).*

Reviewer comment: Sup Fig 4b. I am not sure I understand the significance of these plots. What point are you making?

*Author response: To reduce potential experimental bias and improve confidence in the results, we performed 3 biological replicates of STARRseq. The plots in Supplementary Figure 2B (previously Supplementary Figure 4B) demonstrate that there is a good reproducibility between the individual replicates at both control and ARBS. We chose to include this figure as earlier STARRseq plasmids (PMID: 27565349) were notorious for high variability and many authors would test >3 biological replicas. We have better described these plots in the Supplementary Figure 2 legend.*

Reviewer comment: Figure 3. You state that the peak height of AR is the best predictor. Does this mean that the AR ChIP-seq peak set you started with may have not been of great quality, containing many small (false positive?) peaks? Perhaps a high false positive ChIP-seq dataset explains your statement "From this we found that only a fraction of ARBS (7%) showed androgen-dependent enhancer activity, while the majority (81%) were inactive"?

*Author response: See our description above of how the ChIPseq peaks were selected. The conservative selection criteria used should greatly limit the number of false positive peaks. We feel confident in the quality of these ARBS as they were found in all AR cistromes of primary PCa (n=13) or normal prostate tissue (n=7) (PMID: 26457646). The PCa AR binding sites were subsequently confirmed in a larger clinical population (n=97)(PMID: 30464211). Overall, the ARBS tested represent very high-confidence clinical binding sites.*

Reviewer comment: Basically, I don't think that figure 4 provides much insight into how to identify a critical enhancer. The results obtained after inactivation of the different enhancer types are not consistent.

*Author response: To validate the impact of different enhancer classes on gene transcription we selected those androgen regulated genes with either simple (single enhancer) or complex (multiple enhancer) ARBS interactions. Using CRISPRi to systematically inactive these CRE, we found that every inducible AR enhancer that we tested, including one located >2Mb from its target gene, was critical for AR-mediated transcription. In contrast some, but not all, of constitutive enhancers or inactive ARBS contributed to AR-mediated gene transcription. These results highlight the complexity of gene regulation where multiple CRE interact with a single gene to drive transcription. In our model of activity the inducible enhancers act as a central “hub” with increased interactions with other ARBS and gene promoters. This does not discount a potential role of other ARBS. Additionally we see equivalent evolutionary conservation at inducible, inactive and constitutively active enhancers suggests that all classes of ARBS may potentially contribute to gene transcription. The text has been changed to better reflect this (page 9).*

Reviewer comment: Fig 6D. You do not define NT and you don't really explain what the experiment was that you have shown in the figure.

*Author response: We thank the reviewer for identifying this. The expression of ZBTB16 after CRISPRi inhibition of its enhancer were normalized to NT (non-targeting) control. We have described this and dramatically expanded the experimental detail in our figure legends (page 32).*

Reviewer comment: For the CRISPRi experiments, did you monitor the genomic region to see if the expected epigenetic modifications occurred?

*Author response: Due to the large number of gRNA used we did not validate every CRISPRi knockdown with epigenetic ChIP-PCR. However, the epigenetic modifications caused by CRISPRi have been experimentally confirmed in numerous publications (PMID: 31053162, 28964699, 31980609, and 26501517). Demonstrating that the system was working we observed strong inhibition with our positive control (gene promoter) in each assay.*

Reviewer comment: You make this statement in the Discussion: "By systematically testing all clinical ARBS for enhancer activity, this provides the first detailed "map" needed to investigate these clinically important problems.

a) I note that you have only done these experiments in one prostate cancer cell line. Because of the very low rate of identification of "functional" enhancers in your experiment, it could easily be that you would get a completely different set if you used a different prostate cancer cell line. Also, what if you used a second promoter, would you have identified a different set of functional enhancers? If so, you do not yet have a detailed map.

b) As stated earlier, the composition of your starting set of ARBS will affect the percentages in your functional experiments. You need to be sure that you have a high quality, comprehensive set of AR ChIP-seq data. It is really difficult to know if this is the case from your description of the datasets. The fact that the height of the AR peak is the best predictor may indicate that you have poor quality peaks in your set of peaks.

*Author response: We agree with the reviewer that there may be differences in the specific AR enhancer activity if tested in different cell lines. However, the strong correlation observed from our single cell line and enhancer associated histone marks in clinical samples suggests that these changes would likely be very subtle. This also suggests that the use of different core-promoters in STARRseq would not significantly change the activity. A recent study demonstrated that use of alternative core promoters does not significantly alter the enhancer activity in STARRseq, more problematic, can act as a conflicting promoter with the bacterial ORI thereby causing confounding false positives and negatives (PMID: 29256496). To avoid these limitations we have done this ARBS screen with the most recent STARRseq system that utilizes the bacterial ORI as the core promoter. As for the quality of the AR peaks tested, we used a very conservative selection criteria and are confident that these represent high-confidence peaks (see selection criteria above). We have better described our selection process in the text (page 4).*

**Second round of review**

**Reviewer 1**

The authors have dealt with my previous criticism in a satisfactory fashion.

**Reviewer 2**

In general, the authors have done a good job at explaining the public datasets that were used and their experimental methods. They have cleaned up the text and the paper is now a lot easier to read and understand. However, I still feel that the paper needs an additional analysis to increase the significance of their finding.

The authors have clearly shown that the there is only a small number of AR binding sites that have androgen dependent activity (using STARR-seq), as compared to a much larger number of genomic binding sites for AR that are either constitutively active or inactive in the STARR-seq assay. They have also found that AR peak height was the best (but not perfect) indicator of predicting if an AR binding site would be an inducible site. However, the significance of these findings is not yet clearly shown.

An important experiment that would demonstrate that their approach is generally useful for identifying the few “functional” TF binding sites from the large set of “non functional” TF binding sites would be to perform a carefully designed set of CRISPRi experiments. In figure 4, they show the results of targeting 4 inducible AR sites, 3 constitutive AR sites, and 2 inactive sites; of these are from only 4 genes. The results are a bit confusing and not quantitatively compared. I think that they should perform a more quantitative analysis, picking 10 each of the 3 sets of AR binding sites (inducible, constitutive, inactive), targeting them via CRISPRi, and then reporting the fold effect on the nearest AR-regulated promoter. Do they, in fact, see a more robust effect on regulation when the inducible sites are targeted? It would be interesting to see a quantitative analysis of the AR peak height vs the results of these experiments. If they could show that they had a statistically significant better chance of identifying a functional site (as evidenced by transcription from the endogenous gene) when using data from their STARR-seq method to classify the AR binding site, this would greatly increase the significance of the paper.

**Authors Response**

**Point-by-point responses to the reviewers’ comments:**

Reviewer #1: The authors have dealt with my previous criticism in a satisfactory fashion.

*Author response: We would like to thank the Reviewer for all of their helpful feedback.*

Reviewer #2: In general, the authors have done a good job at explaining the public datasets that were used and their experimental methods. They have cleaned up the text and the paper is now a lot easier to read and understand.

However, I still feel that the paper needs an additional analysis to increase the significance of their finding. The authors have clearly shown that the there is only a small number of AR binding sites that have androgen dependent activity (using STARR-seq), as compared to a much larger number of genomic binding sites for AR that are either constitutively active or inactive in the STARR-seq assay. They have also found that AR peak height was the best (but not perfect) indicator of predicting if an AR binding site would be an inducible site.

However, the significance of these findings is not yet clearly shown. An important experiment that would demonstrate that their approach is generally useful for identifying the few “functional” TF binding sites from the large set of “non functional” TF binding sites would be to perform a carefully designed set of CRISPRi experiments. In figure 4, they show the results of targeting 4 inducible AR sites, 3 constitutive AR sites, and 2 inactive sites; of these are from only 4 genes. The results are a bit confusing and not quantitatively compared. I think that they should perform a more quantitative analysis, picking 10 each of the 3 sets of AR binding sites (inducible, constitutive, inactive), targeting them via CRISPRi, and then reporting the fold effect on the nearest AR-regulated promoter. Do they, in fact, see a more robust effect on regulation when the inducible sites are targeted? It would be interesting to see a quantitative analysis of the AR peak height vs the results of these experiments.

If they could show that they had a statistically significant better chance of identifying a functional site (as evidenced by transcription from the endogenous gene) when using data from their STARR-seq method to classify the AR binding site, this would greatly increase the significance of the paper.

*Author response: We would like to thank the reviewer for their suggestions. While we observed that only a small number of androgen receptor binding sites (ARBS) have androgen dependent enhancer activity, our data does not suggest that the remaining inactive and constitutive ARBS have no functional role in gene regulation. We have clarified the text to better reflect this and included additional experimental data in support of our findings.*

*In our manuscript we demonstrated that the functional classification generated from STARRseq strongly correlates with enhancer-associated features both in vitro and with clinical samples. We showed that these inducible enhancers act as a central “hub” that increase contacts with both other ARBS and gene promoters. However, while inducible enhancers play a central role, the inactive or constitutive enhancers can also be involved in gene transcription. Once the AR binds to DNA there is a complex interplay between multiple cis-regulatory elements, co-regulatory proteins and mediator complex involving chromatin looping and gene promoters. Altering any one of these features could affect gene transcription. Given this complexity, it is unlikely that only inducible AR enhancers affect androgen-mediated gene transcription. We observed in our CRISPRi experiments that while inducible AR enhancers were required for transcription of all tested genes, those interacting inactive or constitutive enhancers can also affect transcription. This is in agreement with recent studies where both inactive and active enhancers (PMID: 27239026) or low and high transcription factor bind sites (PMID: 26875865) work together to transcribe specific genes.*

*In our previous submission we attempted to argue that all AR binding sites, even those without enhancer activity, can potentially play a role in gene transcription. To better support this, we characterized the androgen-dependent gene transcription and found that those inducible enhancers that interact with other ARBS had significantly increased levels of gene transcription compared to those regions that do not (Figure 4H). Further, in this revision we have better clarified our argument and expanded the discussion section to better highlight the role of minimally active and constitutively active enhancers (pg. 9 and 13).*

**Third round of review**

**Reviewer 2**

The authors have addressed my concerns.
